# Antimony induced structural and ultrastructural changes in *Trapa natans*

**DOI:** 10.1038/s41598-021-89865-2

**Published:** 2021-05-21

**Authors:** Sangita Baruah, Monashree Sarma Bora, Sanghita Dutta, Kalyan Kumar Hazarika, Pronab Mudoi, Kali Prasad Sarma

**Affiliations:** 1 Department of Environmental Science, Tezpur University, Napaam, Tezpur, Assam India; 2 Department of Molecular Biology and Biotechnology, Tezpur University, Napaam, Tezpur, Assam India

**Keywords:** Abiotic, Environmental sciences

## Abstract

Antimony (Sb) is considered as a priority toxic metalloid in the earth crust having no known biological function. The current study was carried out in a hydroponic experiment to study the accumulation of ecotoxic Sb in subcellular level, and to find out the ultrastructural damage caused by Sb in different vegetative parts of *Trapa natans*. Sb-induced structural and ultrastructural changes of *T. natans* were investigated using scanning electron microscope (SEM) and transmission electron microscope (TEM). Experimental plants were exposed to different Sb(III) treatments: SbT1 (1.5 μmol/L), SbT2 (40 μmol/L) and SbT3 (60 μmol/L). Calculated bioconcentration factor (BCF) and translocation factor (TF) showed that at higher concentration (SbT2, SbT3), *T. natans* is a potent phytoexcluder whereas it can translocate a substantial amount of Sb to the aerial parts at lower concentration (SbT1). SEM analysis revealed Sb-mediated structural changes in the size of stomatal aperture, intercellular spaces and vascular bundles of different vegetative tissues of *T. natans*. TEM results showed subcellular compartmentalization of Sb in vacuole and cell wall as electron dense deposition. This is considered as a part of strategy of *T. natans* to detoxify the deleterious effects under Sb stress conditions. Fourier transform infrared spectroscopy (FTIR) study of plant biomass revealed possible metabolites of *T. natans* which can bind Sb.

## Introduction

Antimony (Sb), one of the toxic elements with no known biological function, is of great environmental concern because it is considered as priority contaminant by USEPA and EU^1,2^. It is ubiquitous in the environment and found in plants, soil, water and sediment due to both natural and anthropogenic activities^3,4^. The concentration of Sb occurs naturally in rocks, soil and water environment in the range of 0.15–2 mg/kg, 0.3–8.6 mg/kg and < 1 µg/L, respectively^5^. However, elevated concentrations of Sb have been reported in different environmental spheres due to geogenic as well as human induced activities^3^. Sb is primarily emitted to the living environment due to anthropogenic activities such as coal-combustion and smelting operations of antimony ore extraction^6^.

Antimonite (SbIII) and antimonate (SbV) are two redox states of the element. Although, under highly oxic natural water/hydroponic solution, Sb(III) is transformed to Sb(V) but organic matter, especially the plant root exudates, may avert the conversion of Sb(III) to Sb(V) to a certain extent^3,7^. The plant roots selectively uptake Sb(III) and Sb(V) transporting either through apoplastic or symplastic pathways from root to stem^8^.

A number of studies have demonstrated that aquaglyceroporin (AQP) channels assist the diffusion of Sb(III) to the plant cell but to date, no SbV-specific transporters have been identified^9^. Kamiya et al*.*^10^ have identified an Sb(III) transporter (nodulin 26-like intrinsic protein NIP 1;1) in *Arabidopsis thaliana* and it is a key determinant of Sb(III) sensitivity in the plant. There is every possibility of bioaccumulation and further entering into the food chain due to higher mobility of Sb in oxidized surface water. Epidemiological studies have shown that elevated and long-term Sb, especially Sb(III) exposure results in human diseases like cancers, damage of kidney and liver, dermatitis, and diseases in the cardiovascular and respiratory system^11,12^.

Previous studies revealed that Sb induces production of reactive oxygen species (ROS) that can be extremely deleterious to plant at an elevated concentration^13^. It is established fact that accumulation of toxic metals/metalloids can lead to deactivation of cellular enzymatic activities which can inhibit normal growth of the plant. Detoxification mechanisms in plants to avoid such metal toxicity include the formation of metal-chelates and sequestered away from the sites of metabolism^14,15^. Antimonite (Sb III) has been proven to be a strong inducer of phytochelatin (PC) accumulation in a broad variety of plants. Phytochelatin-based metal/metalloid sequestration and its vacuolar compartmentalization are generally considered as basic tolerance mechanism under metal/metalloid stress^16^. Ultrastructural changes such as impairment of thylakoid system, plasmolysis, and appearance of cytoplasmic lipid droplets were reported in the lichen *Xanthoria parietina* under Sb stress^17^*.*

Phytoremediation is considered as a cost effective and environmental-friendly plant based green technology for the remediation of toxic metal/metalloid^18^. A wide range of aquatic macrophytes are used for the remediation of toxic metals/metalloids from waste water^19–21^. *Dittrichia viscosa*, *Ceratophyllum* sp., *Cistus ladanifer, Calluna vulgaris*, *Digitalis purpurea*, *Erica umbellata* were documented as promising bioaccumulators of Sb^4,22,23^. To date, some plants such as *Achillea ageratum*, *Silene vulgaris*, *Trifolium pratense* L., *Pteris fauriei*, *Humata tyermanii*, *Pteris ensiformis* Burm. have been identified as potential Sb-hyperaccumulator which can translocate very high amount of Sb (> 1000 mg/kg Sb on dry weight basis) to their aboveground parts^13,24–26^.

**Figure 1:**
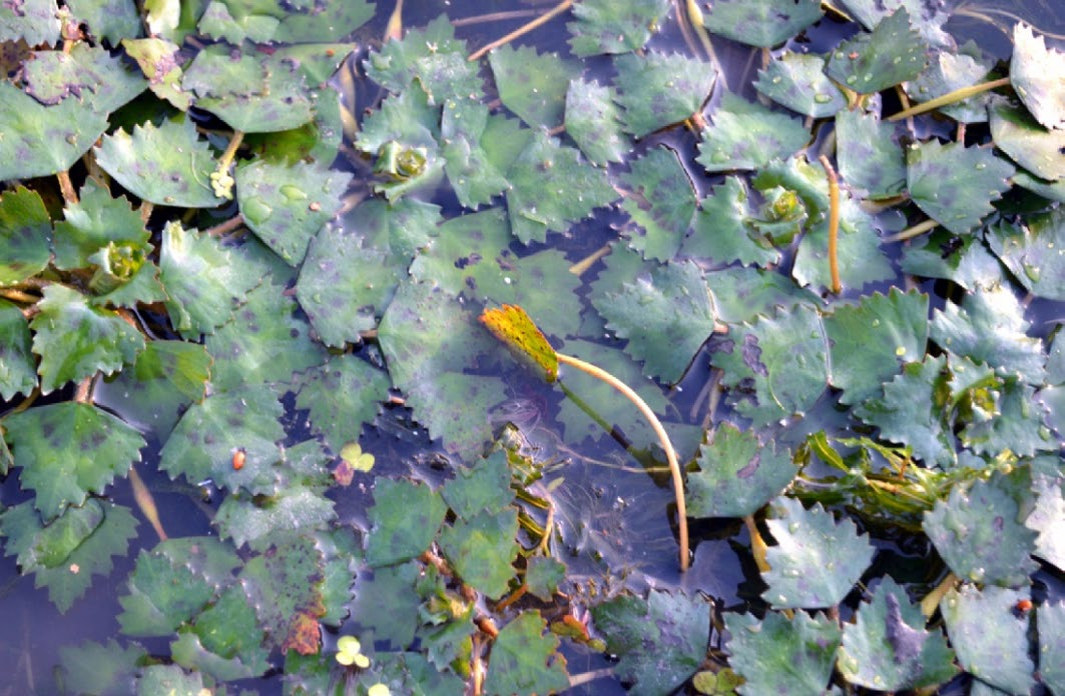
*T. natans* plant in natural condition.

The quick growth, high biomass, profuse root system and easily cultivable are some of the excellent characteristics for which *Trapa natans* (Fig. 1) has been recognised as a prospective plant for phytoremediation and its tolerance to toxic metals/metalloids has been established by many researchers^27–30^. Besides, this plant is rich in hydroxylated polyphenolic compounds (flavonoids, tannins and glycosides) which have potential anti-oxidative activity including the suppression of ROS formation, scavenging ROS and upregulation or protection of antioxidant defence^31^.

Many studies have revealed that plants can uptake and accumulate Sb at high concentration where soil is contaminated with Sb^13,32^. The simultaneous bioaccumulation of both Sb and As by plants have been reported mostly in abandoned mining sites^33,34^. A study on *Pteris vittata* showed that the mechanisms of uptake and translocation of As and Sb differ; As translocates to the aerial parts whereas Sb mostly accumulates in the roots^35^. On contrary to this, other studies showed that maize and *Pteris cretica*, an As-hyperaccumulator, translocate Sb to the aerial harvestable parts under both Sb(III) or Sb(V) exposure^36,37^. Feng et al*.*^36^ proposed Sb deposition in the cell wall and subcellular compartmentalization in the cytosol as the major detoxification mechanism in *P. vittata*. However, there is no published, comprehensive study on the uptake, subcellular localization and ultrastructural changes due to accumulation of Sb in *T. natans*. Therefore, studies on sub-cellular localization and ultrastructural changes in cellular organelles are very important to understand the mechanisms involved in the metal/metalloid tolerance in this plant species. The aims of the present study are: (1) to investigate Sb accumulation and its mode of action to alleviate Sb stress by studying the structural and ultrastructural alterations in *T. natans*; (2) to identify the possible functional group of metabolites that involved in the binding of Sb in the biomass of *T. natans*.

## Results

### Plant responses to Sb toxicity

No visual toxicity symptoms were observed in SbT1 and SbT2 groups of *T. natans*. On contrary to this, chlorosis and fragility in leaf were observed in SbT3 group of plants to a certain extent. In addition, other toxicity symptoms such as browning and twisting of roots were also visible in SbT3 group of *T. natans*.

### Effect of Sb on total chlorophyll content

**Figure 2:**
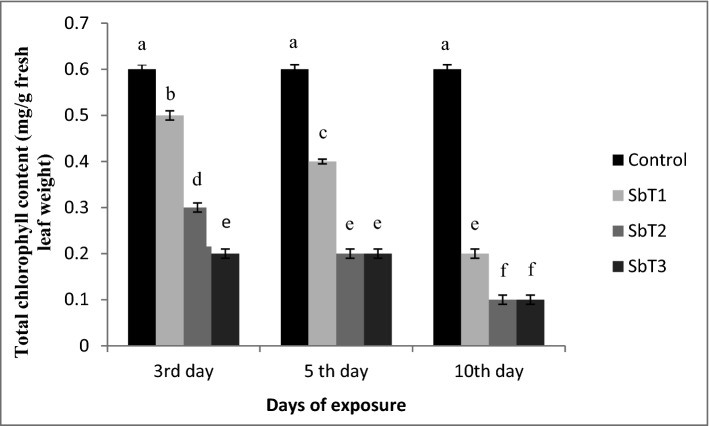
Graphical representation of total chlorophyll content in *T. natans*. Vertical bars represent standard deviation in total chlorophyll content at different initial concentrations. Different letters represent statistically significant differences at *P* < 0.05 from one-way ANOVA and post hoc test (Tukey HSD).

Chlorophyll content is often accepted as an indicator of environmental stress. Significant reduction (*P* < 0.05) in chlorophyll content associated with application of elevated concentration of Sb was observed in the present study. Decreased level of total chlorophyll content with increasing Sb concentration and exposure time was found in the experimental plant species (Fig. 2). This difference in chlorophyll content was significant (*P* < 0.05) according to ANOVA and Tukey HSD test, in *T. natans* grown in different Sb treatments (Supplementary Tables 1, 2).

### Sb concentration in vegetative tissues

Result of Sb concentrations in different plant organs in all three Sb treatments showed that Sb concentrations in roots were somewhat higher (*P* < 0.05) than those in leaves and stems (Table 1; Supplementary Tables 3, 4, 5, 6) except in treatment SbT1 where Sb concentration in stems was slightly higher than roots. Among the three treatments, highest Sb concentrations were observed in leaves (58.60 mg/kg), stems (50.24 mg/kg) and roots (73.66 mg/kg) of treatment SbT2. Plant species exhibiting BCF and TF > 1 is considered as suitable plant for phytoextraction of metals/metalloids^38,39^. In the present study, calculated BCF values for all three Sb treatments were greater than 1 (Table 1). On contrary to this, TF values for all the Sb treatments were found < 1.

**Table 1:** Accumulation of Sb in different vegetative parts of *T. natans.* Data are presented as mean ± SD. Mean values with different superscripts letters within each column or row indicate that they were significantly different at a probability level of 0.05 according to ANOVA and post hoc test (Tukey HSD). Limit of detection (LOD) is 3 µg/L.

Sb treatment	Desired Sb concentration (μmol/L)	Actual Sb concentration (μmol/L)	Sb concentration (mg/kg dry weight)			TF	BCF
			Leaf	Stem	Root		
SbT1	1.5	1.48 ± 0.03	39.78 ± 3.30^a^	47.97 ± 3.67^a^	45.94 ± 2.92^a^	0.96	255.22
SbT2	40	42.70 ± 1.69	58.60 ± 2.99^b^	50.24 ± 3.28^a^	73.66 ± 3.71^d^	0.74	14.25
SbT3	60	61.70 ± 1.60	43.36 ± 3.02^a^	16.68 ± 2.45^e^	59.49 ± 3.47^b^	0.50	7.96

### SEM X-ray microanalysis of leaf, stem and root tissues

Leaf, stem and root of Sb treated plant were examined by scanning electron microscope (SEM) along with energy dispersive X-ray (EDX) unit. This electron microscopy study revealed different Sb induced structural alterations of leaves, stems and roots of *T. natans* treated with SbT3.

**Figure 3:**
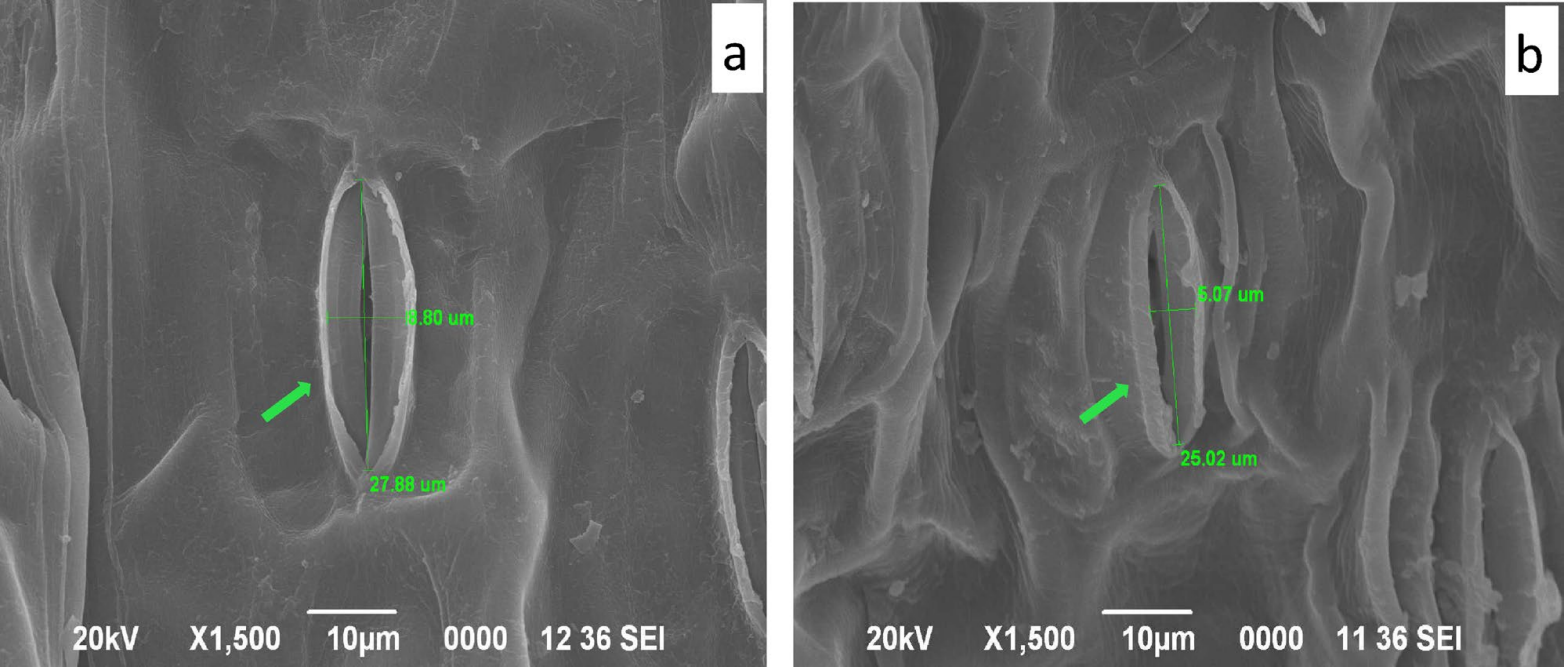
SEM micrographs of abaxial side of *T. natans* leaf: (**a**) leaf epidermis showing stomata in control leaf (**b**) leaf epidermis showing stomata in Sb treated leaf.

Decreased size of stomatal aperture was evident from the SEM micrographs of the abaxial side of Sb treated leaf (Fig. 3b, Supplementary Fig. 1b) with respect to the control leaf (Fig. 3a, Supplementary Fig. 1a). There was a significant difference of 3.73 µm in the stomatal diameter of control (8.80 µm) (Fig. 3a) and Sb treated leaves of *T. natans* (5.07 µm) (Fig. 3b). In EDX spectra of the control plant leaf (Supplementary Fig. 1c) Sb was absent whereas presence of Sb was confirmed in the EDX spectra of Sb treated plants (Supplementary Fig. 1d).

**Figure 4:**
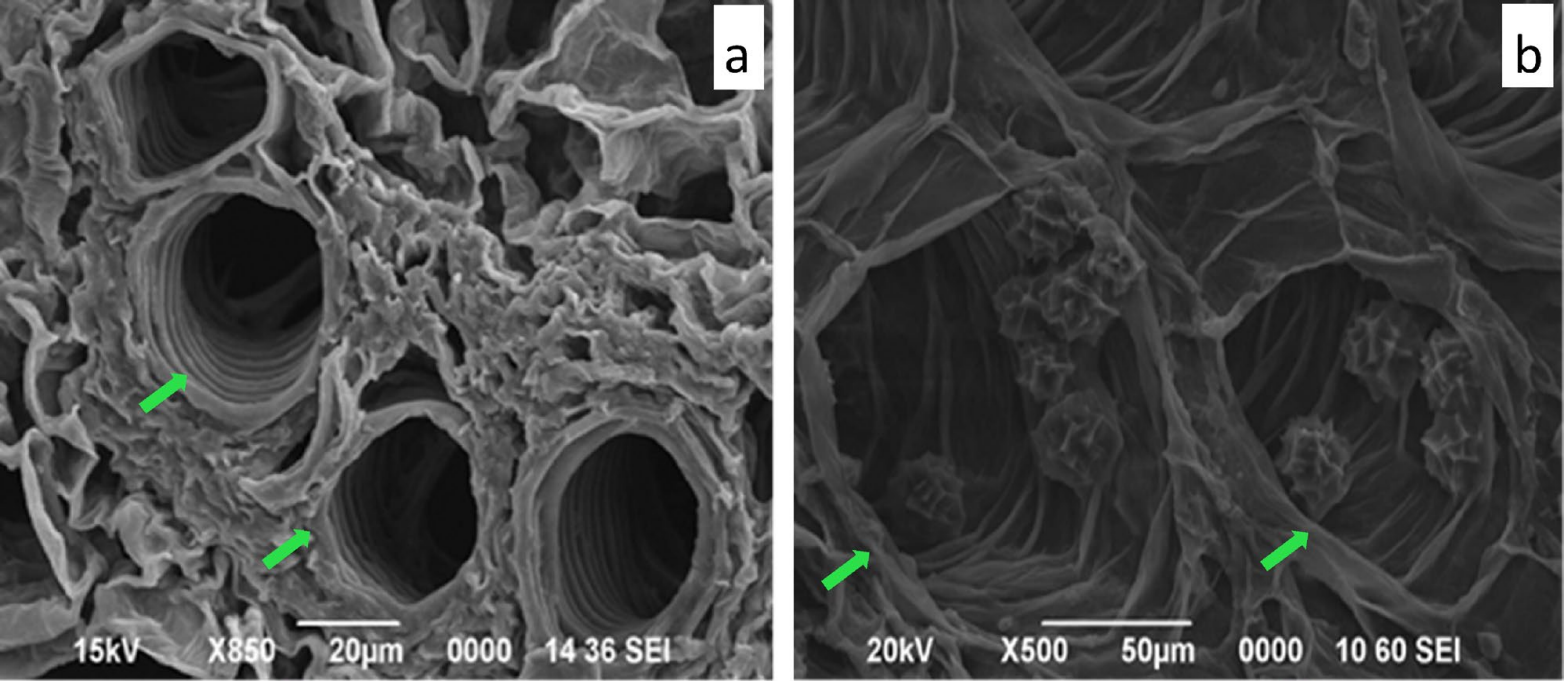
SEM micrographs of transverse section of *T. natans* stem: (**a**) control stem showing well-preserved vascular bundles, (**b**) Sb treated stem showing changes in vascular bundles.

The SEM micrograph of stem of control plant group did not show any abnormality of the vascular bundles (Fig. 4a). Loss of shape and partial collapse of the xylem vessels in the SEM micrograph of treated stem confirmed Sb toxicity in *T. natans* (Fig. 4b). However, cell walls of xylem vessels were not collapsing completely. The EDX spectra clearly showed absence of Sb in transverse section of control stem (Supplementary Fig. 2), whereas Sb weight percentage of 1.20, 1.97 and 0.45 were found in epidermis, cortex and metaxylem vessels, respectively (Supplementary Fig. 3).

**Figure 5:**
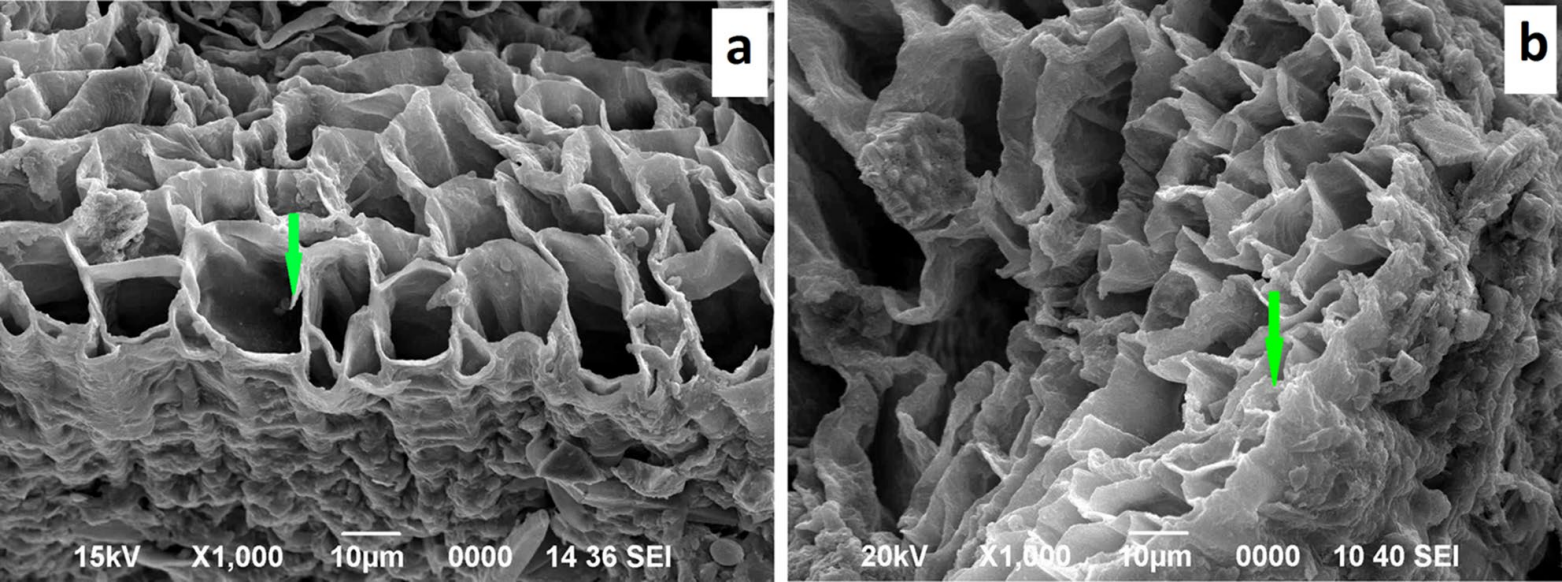
SEM micrographs of transverse section of *T. natans* root: (**a**) control root, (**b**) Sb treated root showing changes in shape of parenchyma cells.

In comparison to the control plants (Fig. 5a), the roots of Sb treated *T. natans* plants showed damages of parenchyma cells (Fig. 5b) which lead to the alteration of cell shape and reduction of intercellular spaces. Metaxylem vessels were uniform in control roots; however, in treated roots metaxylems were partially collapsed resulting in loss of cell shape (Supplementary Fig. 4). Absence of Sb in the control root was confirmed by EDX spectra (Supplementary Fig. 5). However, the EDX spectra of treated root revealed highest enrichment of Sb in the epidermis (1.55 wt%) followed by cortex (0.52 wt%) and metaxylem vessels (0.12 wt%) (Supplementary Fig. 6).

### TEM analysis of leaf and root tissues

**Figure 6:**
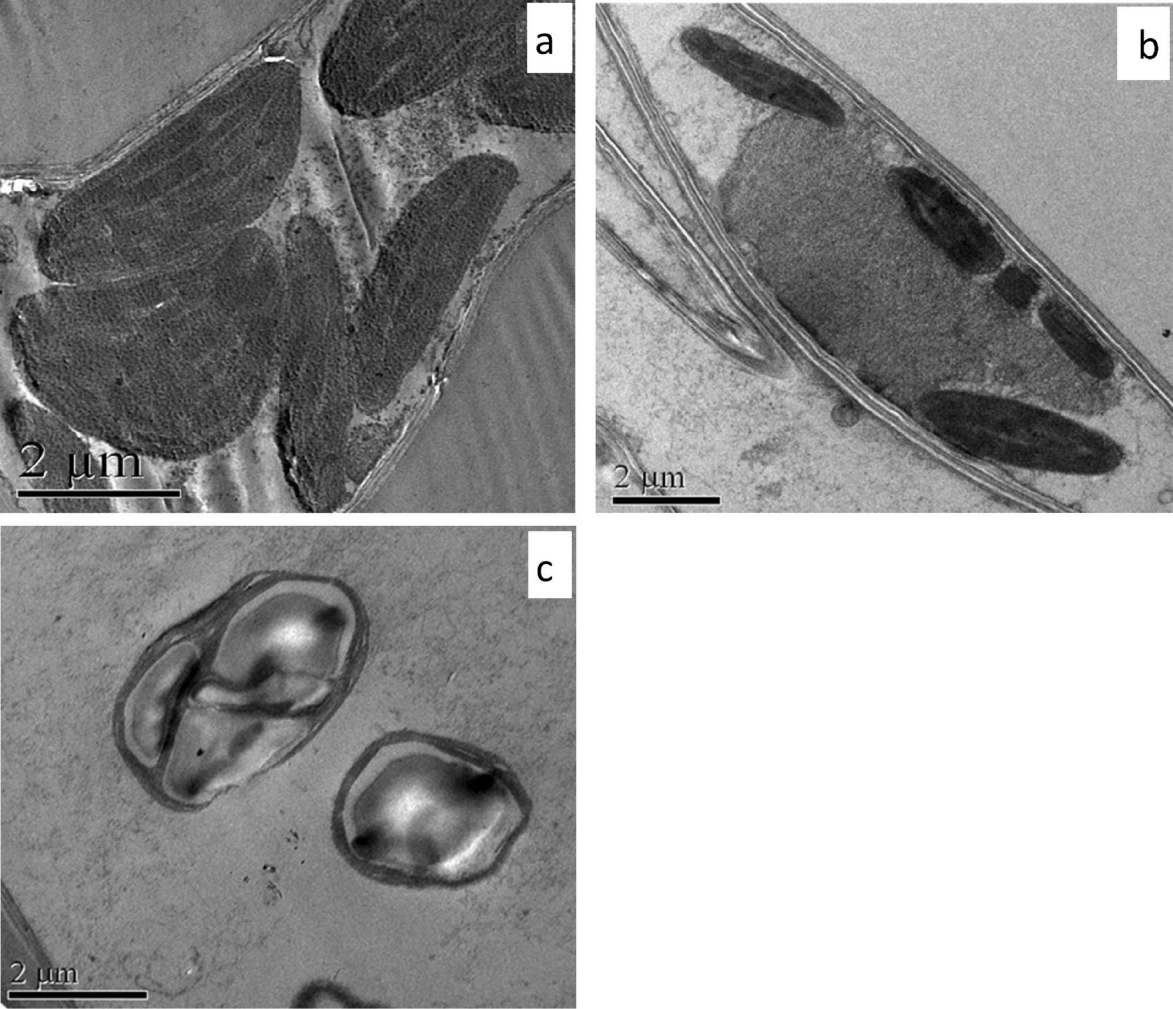
TEM micrographs of *T. natans*: (**a**) a young mesophyll cell of control plant showing numerous chloroplasts, (**b**) damaged and disintegrating chloroplasts in Sb treated plant, (**c**) disturbance in the orientation of the grana and starch accumulation in Sb treated leaf.

The changes in the chloroplast structure of leaf cells of Sb treated *T. natans* were examined by transmission electron microscopy (TEM). The TEM micrographs of control leaf cells of the plant showed no abnormality or disorganization in the ultrastructural view of the chloroplast (Fig. 6a). But, the chloroplasts of the Sb treated plant cells showed disintegration of the inner membrane, disorganization of the structures of grana and stroma, and accumulation of starch (Fig. 6b,c). Ellipsoidal shapes of chloroplasts were observed in the treated plant cells (Fig. 6c).

**Figure 7:**
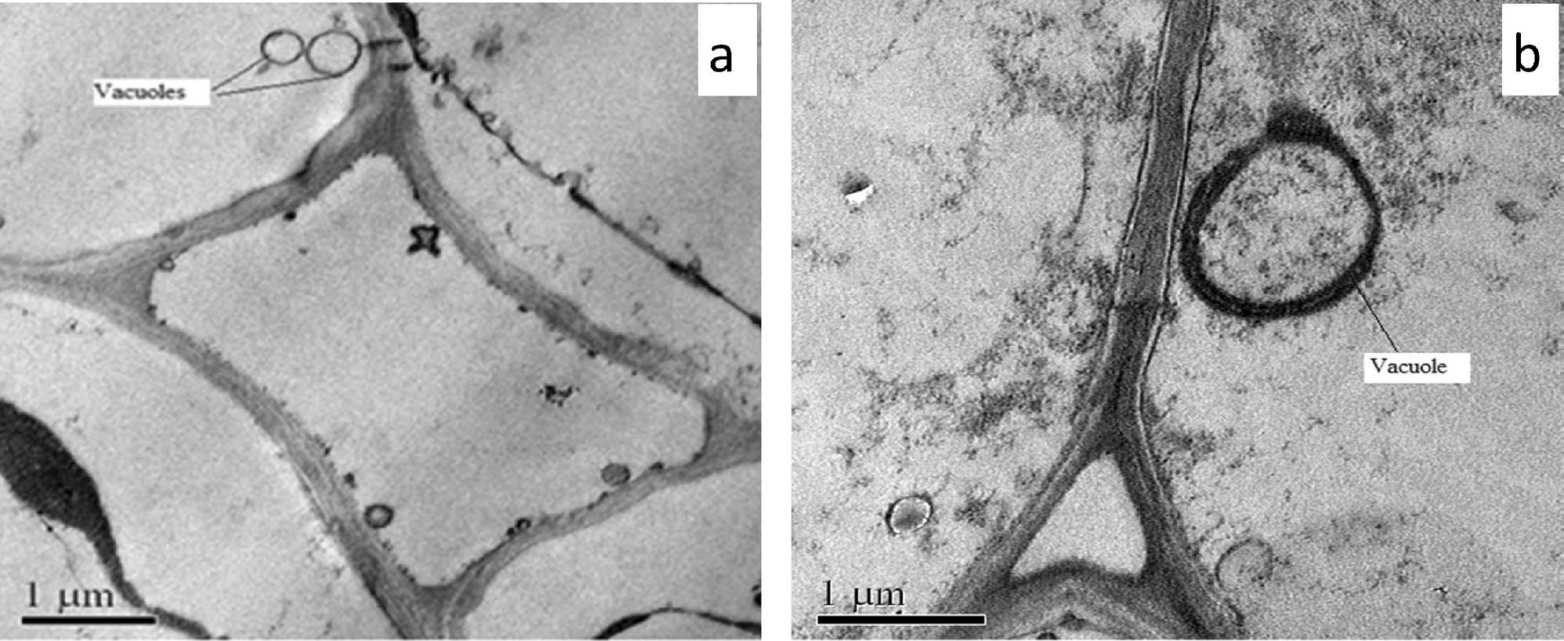
TEM micrographs of transverse section of roots of *T. natans*: (**a**) control plant root showing the vacuoles, (**b**) Sb treated plant root showing metal/metalloid precipitation within the vacuole.

**Figure 8:**
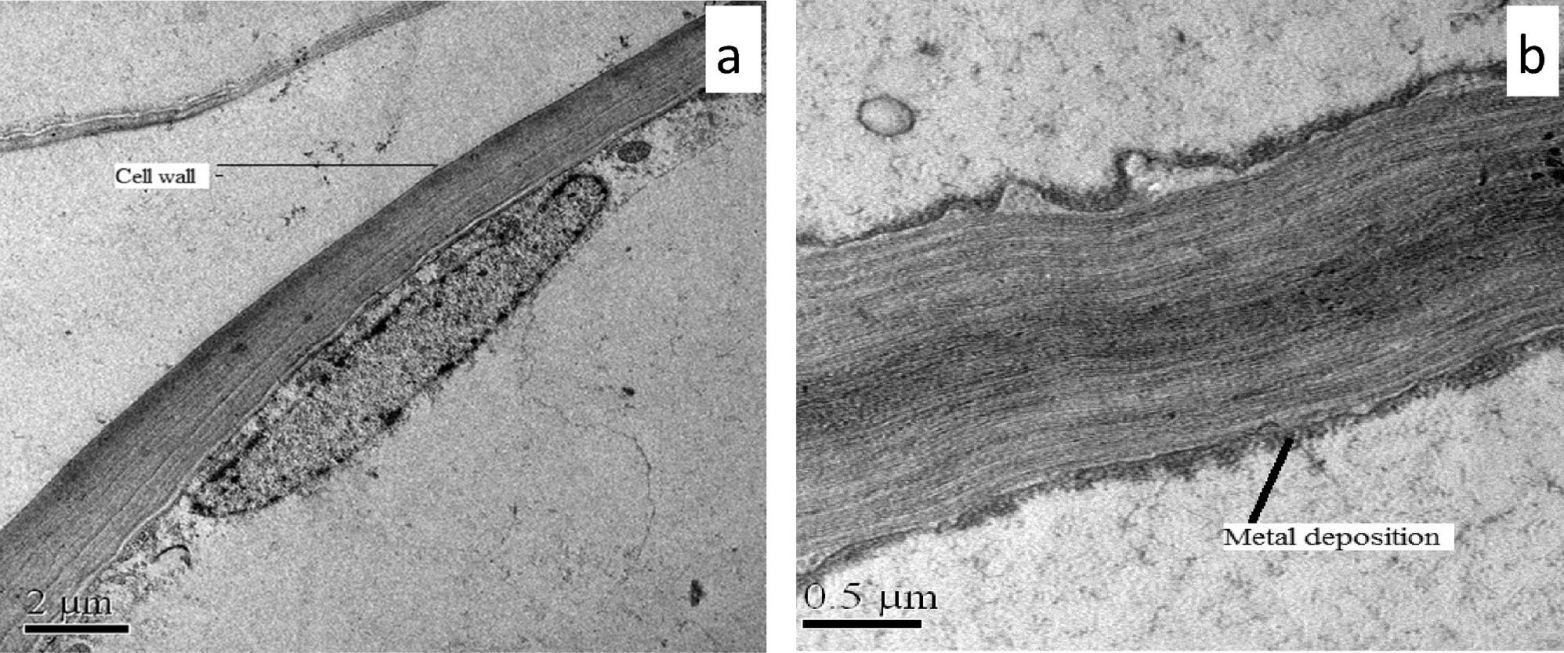
TEM micrographs of root cells of *T. natans*: (**a**) cell wall of control plant, (**b**) Sb treated cell wall showing numerous black deposits.

Several small vacuoles (without metal/metalloid deposition) were found in mature root cells of control plant (Fig. 7a) whereas, the TEM micrograph of Sb treated root cell showed presence of vacuoles with electron dense precipitation (Fig. 7b). The cell wall of root cell did not exhibit any ultrastructural alteration in control *T. natans* (Fig. 8a). On contrary to this, some electron dense precipitates were observed in the intercellular spaces of the root cells which are generally adsorbed on the cell walls (Fig. 8b).

### Fourier transform infrared spectroscopy (FTIR) analysis

**Figure 9:**
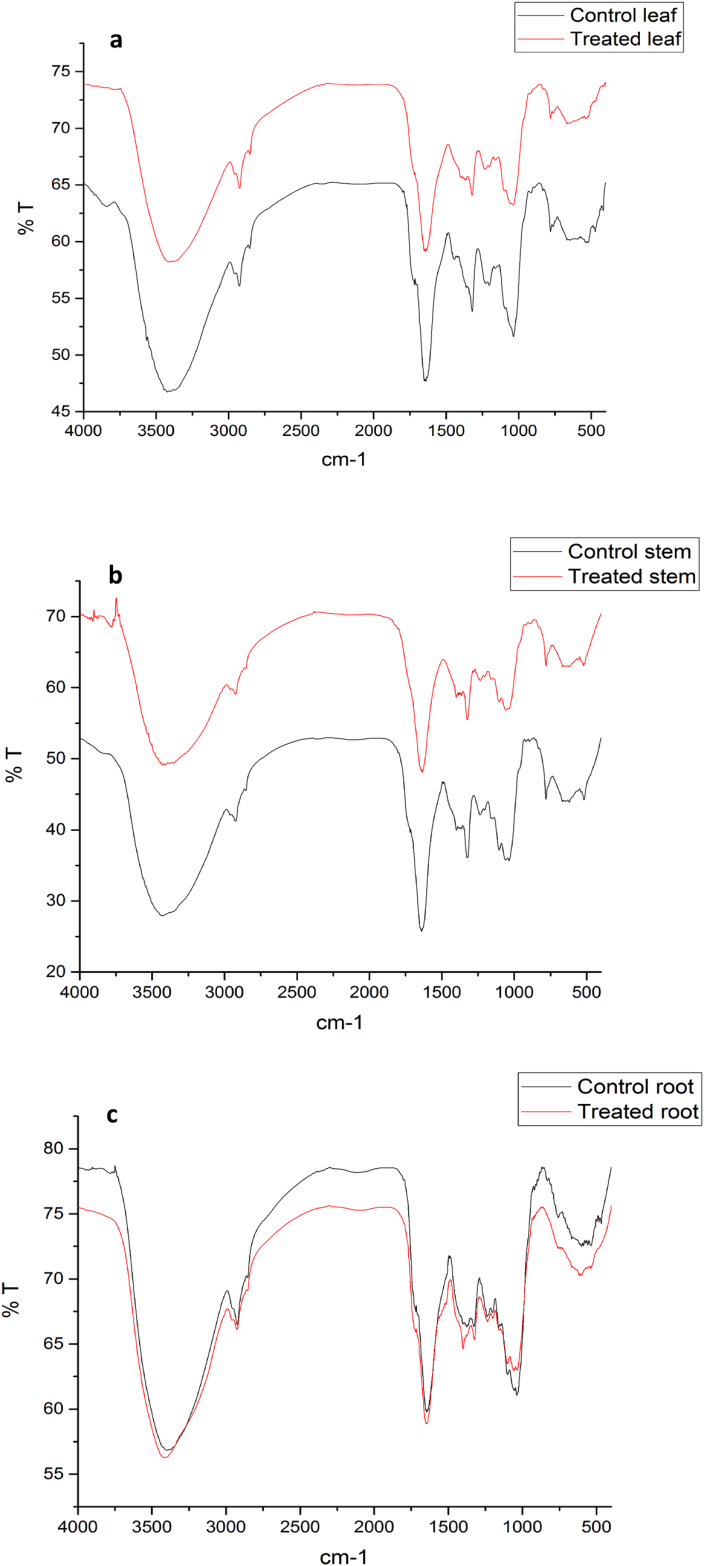
FTIR spectra of control and Sb treated biomass of *T. natans*: (**a**) control and Sb treated leaf, (**b**) control and Sb treated stem, (**c**) control and Sb treated root.

FTIR spectra of the control and Sb treated plant biomass of *T. natans* are presented in Fig. 9 which displayed a number of absorption peaks in the region of 400–4000 cm^−1^. Shifting of the peak position in the FTIR spectra of the Sb-loaded biomass with reference to that of the control plant biomass indicates the binding of Sb with different functional groups present in the biomass (Table 2).

**Table 2:** Characteristic infrared absorption bands of various metabolites in the biomass of *T. natans. CL* control leaf, *TL* treated leaf, *CS* control stem, *TS* treated stem, *CR* control root, *TR* treated root.

Wave number (cm^−1^)		Functional group assignment	References
Peak position in control biomass	Peak position in Sb treated biomass		
3422.24 (CL)	3399.88 (TL)	–OH group of saponin and tannin	Almutairi and Ali^40^; Socrates^41^
3430.43 (CS)	3412.55 (TS)		
3395.41(CR)	3412.52 (TR)		
2925.85 (CL)	2928.66 (TL)	C–H group of saponin	Almutairi and Ali^40^
2925.55 (CS)	2925.25 (TS)		
2926.00 (CR)	2925.25 (TR)		
1636.24 (CL)	1645.92 (TL)	C=O group of flavonoids Amide I (C=O stretching, C–N stretching, N–H bending vibration)	Heneczkowski et al*.*^43^; Yu^46^; Yu^47^
1637.73 (CS)	1635.49 (TS)		
1646.67 (CR)	1648.90 (TR)		
1401.53 (CS, CR)	1401.53 (TS, TR)	C–H bending vibrations from asymmetric CH_3_ of lipid, polysaccharides and cellulose	Dokken and Davis^42^
1319.57 (CL)	1321.80 (TL)	–C–OH deformation vibration of flavonoids	Heneczkowski et al*.*^43^
1321.80 (CS)	1326.27 (TS)		
1326.27 (CR)	1324.04 (TR)		
1201.11, 1228.67 (CL)	1203.33, 1233.88 ( TL)	Asymmetric C–O–H deformation of hemicelluloses and lignin	Dokken and Davis^42^
1203.33, 1233.88 (CS)	1204.08, 1235.37 (TS)		
1204.08, 1237.61 (CR)	1199.61, 1235.37 (TR)		
1038.67 (CL)	1037.18 (TL)	C–O–C (oligosaccharide linkage) of saponin; C–C, C–O stretching of carbohydrates	Almutairi and Ali^40^; Wang et al*.*^44^
1059.53 (CS)	1057.29 (TS)		
1055.06 (CR)	1059.52 (TR)		
783.11 (CL)	782.35 (TL)	–C–OH stretching vibrations of flavonoids	Heneczkowski et al*.*^43^
781.61 (CS)	783.84 (TS)		
757.02 (CR)	759.25 (TR)		
646.75 (CL)	669.11 (TL)	C‒S stretching of disulphide	Coats^45^
621.41 (CS)	619.18 (TS)		
601.29 (CR)	605.76 (TR)		
537.22 (CL)	532.75 (TL)	Alkyl halide	Coats^45^
518.59 (CS)	520.82 (TS)		
536.47 (CR)	538.71 (TR)		

The peaks in the regions of 3395.41–3430.43 cm^−1^ (broad and most intense), 2925.25–2928.66 cm^−1^ (medium), 1401.53 (weak), 1319.57–1326.27 cm^−1^ (medium), 1199.61–1237.61 cm^−1^ (weak), 1037.18–1059.53 cm^−1^ (strong), 757.02–783.84 cm^−1^ (weak), 601.29–669.11 cm^−1^ (weak) and 518.59–538.71 cm^−1^ (weak) were ascribed to –OH stretching vibration (tannin and saponin)^40,41^, C–H stretching vibration (saponin)^40^, C–H bending vibration (asymmetric CH_3_ of lipid, polysaccharides and cellulose)^42^, –C–OH deformation vibration (flavonoids)^43^, asymmetric C–O–H deformation (hemicellulose or cellulose)^42^, C–O–C oligosaccharide linkage group (saponin)^40,44^, –C–OH stretching vibrations (flavonoids)^43^, C–S stretching vibrations and alkyl halide groups^45^, respectively. Furthermore, sharp and intense peaks in the region of 1635.49–1648.90 cm^−1^ revealed the presence of flavonoid (C=O stretching) and amide I (C=O stretching, C–N stretching, N–H bending)^43,46,47^. This comparison of the FTIR spectra of control and Sb-treated biomass (Table 2) showed shifts in some spectral regions responsible for functional groups such as O–H, C–H, N–H, C=O, C–O–H, C–O–C, C‒S which confirm their involvement in binding of Sb in *T. natans*. However, in certain region of the spectra no shifting of peak was observed.

## Discussion

### Plant responses to Sb toxicity

Chlorosis of the leaves was observed in Sb treated *T. natans* plants (SbT3), which is considered as one of the most predominant symptoms of toxic metal/metalloid phytotoxicity^15^. The chlorophyll content of *T. natans* was found to be decreased with the increase in Sb concentration. Pan et al*.*^48^ reported the impact of Sb on chlorophyll content and the photosynthetic efficiency of maize treated with Sb concentration of 10, 50, 100, 500 and 1000 mg/kg in the soil, and found that both parameters were negatively influenced only at higher concentrations. The cause of decrease in chlorophyll level could be due to the inhibition of two enzymes of chlorophyll biosynthesis, namely ferredoxin NADP^+^ reductase and δ-aminolevulinic acid dehydratase (δ-ALAD)^49^. Liu et al*.*^50^ documented that enhanced chlorophyllase action can inhibit photosynthesis in plants under stress conditions.

### Sb concentration in vegetative tissues

Calculated BCF (255.22) and TF (0.96) values for *T. natans* grown in treatment SbT1 evinced that although it falls in the category of phytostabilizer, a significant amount of Sb is translocated to shoots. However, as the initial concentration increased, TF values for treatments SbT2 (0.74) and SbT3 (0.50) decreased where the plant acts as a potent phytostabilizer, and exclusion of Sb from aerial vegetative tissues can be considered as Sb tolerant strategy of the plant species. Besides translocation of Sb from root to shoot, certain amount of Sb may have directly absorbed by the leaves of *T. natans* as the leaves are in contact with the solution. For example, Maine et al*.*^51^ also reported that the direct contact of leaves with metal solution is the main cause of increase of chromium in the aerial parts of some floating macrophytes, although they are poor translocator of the metal from roots to the aerial parts. Variation of translocation factor (TF) and enrichment factor (EF) with metal concentration has been reported by Liu et al*.*^52^ Literature studies showed that quite a few plants such as *Hygrophila auriculata* and *Cyperus exaltatus* (Pb), *Sphaeranthus gomphrenoides*, *Pluchea dioscoridis* and *Cyperus articulates* (Cd), *Sphaeranthus gomphrenoides*, *Typha capensis*, *Pluchea dioscoridis* (Ni) were identified as potential excluders of metals/metalloids from their substrate^53^.

### Sb-induced structural and ultrastructural alterations

Stomatal closure is one of the key molecular and physiological responses of plants to restrict water loss when plants are in stress. Sb toxicity causes stomatal closure in the leaves of the experimental plant species. Inadequate concentration of carbon dioxide via stomatal closure leads to photosynthesis inhibition^54^. An intricate system of signaling pathways regulates stomatal closure, where abscisic acid (ABA) plays the major role in association with jasmonic acid (JA), cytokinins, ethylene, and auxins^55^. Daszkowska-Golec and Szarejko^56^ also reported that shrinkage of guard cells under stress conditions leads to the stomatal closure. In the present study, a significant damage to metaxylem vessels was observed. Damage of vascular bundle, especially the xylem vessels, is of great concern as it forms an integrated network that connects all parts of the plant and is a principal water conducting tissue. Kasim^57,58^ also reported decrease in diameter of metaxylem vessel in vascular bundle of leaf midrib which led to reduction in shoot growth in Zn treated *Phaseolus vulgaris* and, Cu and Cd treated *Sorghum bicolor*. However, there is contradicting report in this regard where increase in the diameter of metaxylem vessel was also observed in roots of *Matricaria chamomilla* during early exposure days of Pb treatment^59^. The most obvious consequence of the decrease in size of the metaxylem is the reduction of upward movement of water and mineral from root to shoot. The ultrastructural changes caused due to Sb in the vascular bundle may also modify the water status of leaves^60^, indicating declining of water level in leaves which in turn leads to stomatal closure. Moreover, researchers have reported toxic effects of metal in the epidermis and parenchyma cells in the cortex of root and stem in the form of disintegration of cells, loss of shape and size^57,58^. In our study, the highest weight percentage of Sb was observed in the epidermis region of stem. Similar result of very high concentration of Ni in epidermis area has also been reported in shoots of *Alyssum inflatum*^61^. Detection of Sb in the xylem vessels of root and shoot suggests transportation of Sb through xylem vessels from root to aerial parts. Although the shapes of the metaxylem vessels changed under Sb stress in the present study, xylem cell walls were remained intact which can be attributed to the heavily lignified composition of cell walls for providing mechanical strength as well as preventing collapse of vessels.

Integrity of chloroplast ultrastructure is necessary for normal performing of photosynthesis in the plant cell as the whole process of photosynthesis is completed in the chloroplasts. Our experimental results revealed disorganization of chloroplast ultrastructure in Sb treated plants. Researchers have shown that toxic metals/metalloids affect the chloroplast structure by degradation of grana and stroma lamellae along with enhancement of the quantity and dimension of plastoglobuli^62^. Starch accumulation was also evident within the chloroplast of Sb treated *T. natans*. Accumulation of starch grains in the plastid is one of the common features under metal/metalloid toxicity which may be attributed to the impairment of biochemical processes regarding starch synthesis under Sb stress^63^. Enhanced volume of the stroma and disorganization of the thylakoid membrane results in the change of shape of chloroplasts^64^. Disorganization and looseness of the lamellar structure in chloroplast could cause decrease in the activity and function of photosystem II^65^. Thus, the chloroplast is considered as one of the most targeted sites of trace elements^66^. Despite of severe destructions in this organelle toxic metals/metalloids do not accumulate in the chloroplasts^67^; rather they are found abundantly in the apoplast and the vacuole^68^. However, on contrary to the higher plants, 50–60% Cd was reported in the chloroplast of cell-wall deficient *Chlamydomonas reinhardtii* and plant vacuole deficient *Euglena* gracilis^69,70^. Although, above results indicate capability of chloroplasts to accumulate toxic metals, but generally this is not the target organelle of plant cell having cell-wall and vacuole for accumulation of metal/metalloid. The results of our study also indicate that Sb accumulation in vacuoles is the most effective system for maintaining a very low cytoplasmic Sb concentration in *T. natans*.

Cell wall also played an important role in the storage of metals/metalloids as it acts as the first barrier for entry of metal/metalloid into symplastic compartments of the cell and one of our recent publications also showed that Cd was mainly accumulated in the cell wall^71^. The binding of Sb in the cell wall and Sb compartmentalization in the cytosol have been proposed as possible mechanisms for detoxification in Sb hyperaccumulator ferns like *Pteris cretica* and *Pteris fauriei*^13^. This is consistent with our results as TEM micrographs of the Sb treated root cells of *T. natans* confirmed electron dense deposition of Sb in the vacuoles. As stated earlier, the storage and Sb deposition as fine precipitates in the vacuoles of root cells indicate Sb detoxification mechanism of cell in this experimental plant species preventing high concentration of the metalloid in the cytosol^72^. It is also reported that intracellular ligands such as phytochelatins (PCs) and metallothioneins (MTs) play significant role in the metal detoxification mechanism^73^. Various metal transporter protein families such as ATP-binding cassette (ABC), cation diffusion facilitators (CDF), heavy-metal P-type ATPases (HMA), and natural resistance-associated macrophage proteins (NRAMP) are involved in this detoxification process. The ABC transporters are reported to be involved in toxic metal/metalloid transport into the vacuole and among these transporters, two subfamilies, viz. the multidrug resistance-associated proteins (MRP) and pleiotropic drug resistance (PDR), are mainly active in this process^74^. The observed Sb depositions along the cell wall of the root cells of Sb treated *T. natans* could be considered as one of Sb tolerance mechanisms. The importance of cell wall in binding of toxic metals in plant cell has already been documented^15,75^. The binding of cationic elements to the negative-charged pectic compounds (e.g. galacturonic acid) in cell wall takes place through passive ion exchange process^76^. Under stress conditions breakage of membrane allows binding of more cations in the newly exposed protein sites resulting in higher cation exchange capacity (CEC) in the plant cell^77^.

### Fourier transform infrared spectroscopy (FTIR) analysis

FTIR is important in elucidating the structure and composition of both primary and secondary metabolites which are present in the plant biomass. Primary metabolites mainly proteins, lipids and carbohydrates are essential constituents of plants for normal growth, development and reproduction, while secondary metabolites are generally distinct class of specialized substances to specific plant species^78^. Phytochemical investigation on *T. natans* has led to identification of various primary and secondary metabolites such as carbohydrates, saponins, phytosterol, flavonoids, glycosides, fats and tannins^79^. The formation of varying FTIR spectra in metalloid-loaded plant biomass validated the contribution of different functional groups of metabolites in binding of Sb in the experimental plant biomass. The results of FTIR analysis indicated involvement of functional groups such as O–H, C–H, N–H, C=O, C–O–H, C–O–C, C‒S for Sb binding, which is also supported by previous study^80^. Conclusively, from this infrared spectroscopic analysis different metabolites such as proteins, lipids, carbohydrates and secondary metabolites, especially flavonoids, tannins, saponins are found as possible plant constituents responsible for binding of Sb in the biomass of *T. natans*.

## Conclusions

The present study is an appraisal of the structural as well as ultrastructural alterations in the different organelles of *T. natans* due to subcellular accumulation of Sb. This study will also help to shed some light on the accumulation pattern and ultrastructural modifications of *T. natans* under Sb toxicity. In the electron microscopy studies, Sb was found as electron dense precipitate mainly in cell wall and vacuoles which can be considered as Sb tolerant mechanism of the studied plant species. Besides, accumulation of starch in the chloroplast, disorganization of chloroplast ultrastructure, change of shape of chloroplasts were some of the noticeable changes that were evident in *T. natans* due to Sb stress*.* FTIR analysis confirmed the possible functional groups of various metabolites present in the biomass of the plant species for binding of Sb ions. It is evident from the present study that in general *T. natans* is a suitable plant for phytostabilization of Sb by storing proportionately higher amount of the element in the rhizosphere restricting their mobilization and rendering them harmless. But at low concentration substantial amount of Sb was translocated to the stem and leaf. Although the plant cannot be clearly classified as phytoextractor of Sb at lower Sb concentration (as the TF slightly less than 1), but can be considered as a borderline case. Thus, *T. natans* which is abundantly available in many natural wetlands of Assam can be regarded as one potential candidate for phytoremediation of Sb.

## Materials and methods

### Description of macrophyte

*T. natans* (Water chestnut) is an herbaceous aquatic floating-leaved macrophyte with a rosette of floating leaf belonging to the monogeneric family Trapaceae that grows plentifully in the freshwater lakes of Assam (Fig. 1). The plant is also grown commercially in many parts of India for its nutrient rich edible seeds. *T. natans* of similar size and weight were collected from Joysagar Pond of Sibasagar District, Assam for the experimental purpose and washed thoroughly in running tap water and deionized water to avoid any surface contamination.

### Hoagland’s nutrient solution

The modified Hoagland’s solution was prepared using the following chemicals (in M): Ca(NO_3_)_2_·4H_2_O 5 × 10^−3^, KNO_3_ 5 × 10^−3^, KH_2_PO_4_ 1 × 10^−3^, MgSO_4_·7H_2_O 2 × 10^−3^, FeSO_4_·7H_2_O 0.02 × 10^−3^, H_3_BO_3_ 0.045 × 10^−3^, CuSO_4_·5H_2_O 0.3 × 10^−6^, Na_2_MoO_4_·2H_2_O 0.1 × 10^−6^, MnCl_2_·4H_2_O 0.01 × 10^−3^ and ZnSO_4_·7H_2_O 0.8 × 10^−3^^81^. The floating-leaved *T. natans* plants were acclimatized for 7 days in modified 0.2-strength Hoagland’s nutrient solution (0.2 X HS).

### Preparation of antimony stock solution and experimental design

Antimony (SbIII) stock solution of 100 μmol/L was prepared by dissolving SbCl_3_ (Merck analytical grade) in Milli-Q (18.2 MΩ cm conductivity) water. An outline of the experimental design is presented in Supplementary Fig. 7. The acclimatized plants were transferred to 4 sets of 2 L opaque non-reactive round plastic containers (depth 10 cm and diameter 28 cm) having three tubs for each set. Set I was considered as control group (SbT0) containing 2 L of 0.2X HS without addition of Sb(III) solution. Plants with uniform size were placed in set II, III and IV filled with 0.2 X HS, but supplemented with desired amount of Sb(III) stock solution for making final Sb(III) concentrations of 1.5 μmol/L (SbT1), 40 μmol/L (SbT2) and 60 μmol/L (SbT3), respectively. Plants were allowed to remain in contact with the treatment solutions for 10 days under natural photoperiod and temperature. The pH of the treatment solutions was adjusted to 6.0 with dilute NaOH or HCl. All the treatment groups were arranged in a completely randomized design and three replicates of experimental plant species were set up for each treatment group. After 10 days of experimental period, plants were harvested, washed with Milli-Q water and separated into leaves, stems and roots. The loss of water volume in the containers due to evapo-transpiration was maintained by adding deionized water. A pilot study was conducted in order to design the concentration of the Sb in the experiment. Three different concentrations of Sb(III) were chosen considering the fact of pilot study that the experimental plant species was found to be tolerant to these concentrations to a great extent, although plants exposed to SbT3 showed some visual toxicity symptoms in the study.

### Chlorophyll content measurement

Leaf chlorophyll content of *T. natans* was estimated according to Arnon^82^ on 3rd, 5th and 10th day of the experiment. Fresh plant leaf (0.5 g) was homogenized with mortar and pestle, and extracted in 10 mL of 80% chilled acetone. The absorbance of the extract was recorded in UV–visible spectrophotometer at wavelengths of 645 and 663 nm.

### Analysis for Sb bioaccumulation in plant vegetative tissues

To determine the Sb accumulation efficiency in different parts of *T. natans,* leaves, stems and roots were separated. Drying of harvested plant materials was carried out using standard protocol^21^. The plant materials were digested with a mixture of HNO_3_ (69%) and H_2_O_2_ (30%) in microwave digestion system (Anton Paar Multiwave GO) at 105 °C for 30 min and then cooled for 3 min. Samples were diluted to a final volume of 12.5 mL with deionized water^83^. The blank and certified reference material (SRM-1573a tomato leaves, NIST standard reference material, USA) were used for quality control. Sb concentrations in the digested samples were measured by ICP-OES (Optima 2100, Perkin Elmer) with detection limit 3 µg/L.

### Procedure for microscopic study

For the microscopic study leaf samples from control group (SbT0) and Sb treated group (SbT3) were cut out from the middle portion of the leaf whereas root and stem samples were excised from 2 cm below and above, respectively, the shoot–root intersection.

For scanning electron microscopy analysis (SEM) small pieces of leaves, stems and roots (3–4 mm) were instantly fixed in 3% glutaraldehyde prepared in 0.05 M phosphate buffer for duration of 90 min, followed by secondary fixation in 2% osmium tetroxide prepared in 0.01 M sodium cacodylate buffer for 30 min^84^. Samples were dehydrated in a graded acetone series (30–100%, v/v). SEM photographs were obtained using SEM model JEOL-JSM-6390 LV along with EDX unit, with an accelerating voltage of 15 kV and 20 kV.

The sample preparation protocol for transmission electron microscopy analysis (TEM) involves fixation, sectioning and staining of the sample^85^. Leaf and root samples were fixed in 2.5% glutaraldehyde in 0.05 M potassium phosphate buffer (pH 7.1) for 3 h. Osmium tetroxide was used as a stain and fixative for studying morphology in biological electron microscopy. Then the samples were dehydrated in a graded ethanol series (30–100%, v/v) and embedded in Spurrs epoxy resin. Ultrathin sections of the blocks were obtained using an ultramicrotome. Sections were post-stained with basic lead citrate and uranyl acetate for microscopic examination using JEOL TEM.

### Fourier transform infrared spectroscopy (FTIR) analysis

FTIR spectroscopy was used to elucidate different functional groups responsible for binding of Sb ions in leaves, stems and roots of SbT0 and SbT3 group of *T. natans*. For this purpose, different parts of *T. natans* obtained after experimentation were freeze-dried for 24 h using a laboratory freeze-dryer (LSI LyoLab) to preserve its bioactive components. The leaf, stem and root powder (0.0035 g) of the freeze dried biomass were mixed with KBr (0.5 g) as the base material to form pellets^80,86^ and FTIR spectra (400–4000 cm^−1^) were obtained using FTIR spectrometer (Perkin Elmer Spectrum100). FTIR spectra of plant samples before and after absorption were compared.

### Data analysis

#### Bioconcentration factor (BCF)

Bioconcentration factor (BCF) is a ratio which indicates the capacity of the plant to bioaccumulate a specific metal in roots in regards to metal concentration in the medium^87^, and was calculated using the following formula:

$$ {\text{BCF}} = {\text{Concentration}}\;{\text{of}}\;{\text{metal}}\;{\text{in}}\;{\text{root/Initial}}\;{\text{concentration}}\;{\text{of}}\;{\text{metal}}\;{\text{in}}\;{\text{medium}}. $$

#### Translocation factor (TF)

Translocation factor (TF) was calculated to assess the ability of the experimental plant species for translocation of the metal from the roots to the aerial parts^35^. The following formula was used to calculate TF^88^:

$$ {\text{TF}} = {\text{Metal}}\;{\text{concentration}}\;({\text{shoot}}){\text{/Metal}}\;{\text{concentration}}\;{\text{(root)}}. $$

#### Statistical analysis

All the results of the experiment were presented as mean ± standard deviation (SD) of three (n = 3) replicates. One-way analysis of variance (one-way ANOVA) followed by post hoc test (Tukey’s Honestly Significant Difference test) was performed for all the measured variables using SPSS 23.0 to check the significant difference in the evaluated parameters of the Sb treated plants with respect to the control plants. A probability of 0.05 was considered as significant for evaluation of critical values differences.

## Supplementary Information

Supplementary Figures.

Supplementary Tables.
