# Supplementary material for: Antimony induced structural and ultrastructural changes in Trapa natans
Source: Sci Rep. 2021 May 21;11:10695. doi: 10.1038/s41598-021-89865-2 (PMC8140150; doi:10.1038/s41598-021-89865-2)

**Antimony Induced Structural and Ultrastructural Changes in *Trapa natans***

**­­**Sangita Baruah^1^**·** Monashree Sarma Bora^1^**·** Sanghita Dutta^1^**·** Kalyan Kumar Hazarika^2^**·** Pronab Mudoi^2^**·** Kali Prasad Sarma^1^*

1. Department of Environmental Science, Tezpur University, Napaam, Tezpur, Assam, India
2. Department of Molecular Biology and Biotechnology, Tezpur University, Napaam, Tezpur, Assam, India

Corresponding author: [sarmakp@tezu.ernet.in](mailto:sarmakp@tezu.ernet.in)

Sangita Baruah and Monashree Sarma Bora are co-first author (Both of them contributed equally to this work)

**List of Supplementary Figure Captions**

**Supplementary Figure 1.** SEM-EDX micrographs of leaf of *T. natans*: (a) leaf epidermis of control plant (b) leaf epidermis of Sb treated plant, (c) EDX spectra (along with weight percentage) of control leaf, (d) EDX spectra (along with weight percentage) of Sb treated leaf.

**Supplementary Figure 2.** SEM-EDX spectra (along with weight percentage) of control stem of *T. natans*: (a) EDX spectra of epidermis (b) EDX spectra of cortex region (c) EDX spectra of metaxylem vessels.

**Supplementary Figure 3.** SEM-EDX spectra (along with weight percentage) of Sb treated stem of *T. natans*: (a) EDX spectra of epidermis (b) EDX spectra of cortex region (c) EDX spectra of metaxylem vessels.

**Supplementary Figure 4.** SEM micrographs of metaxylem vessels in control (a) and Sb treated (b) *T. natans*.

**Supplementary Figure 5.** SEM-EDX spectra (along with weight percentage) of control root of *T. natans*: (a) EDX spectra of cortex region (c) EDX spectra of metaxylem vessels.

**Supplementary Figure 6.** SEM-EDX spectra (along with weight percentage) of Sb treated root of *T. natans*: (a) EDX spectra of epidermis (b) EDX spectra of cortex region (c) EDX spectra of metaxylem vessels.

**Supplementary Figure 7.** Diagrammatic representation of the experimental design. n denotes the number of replicates cultured for each treatment.

**Supplementary Fig. 1**


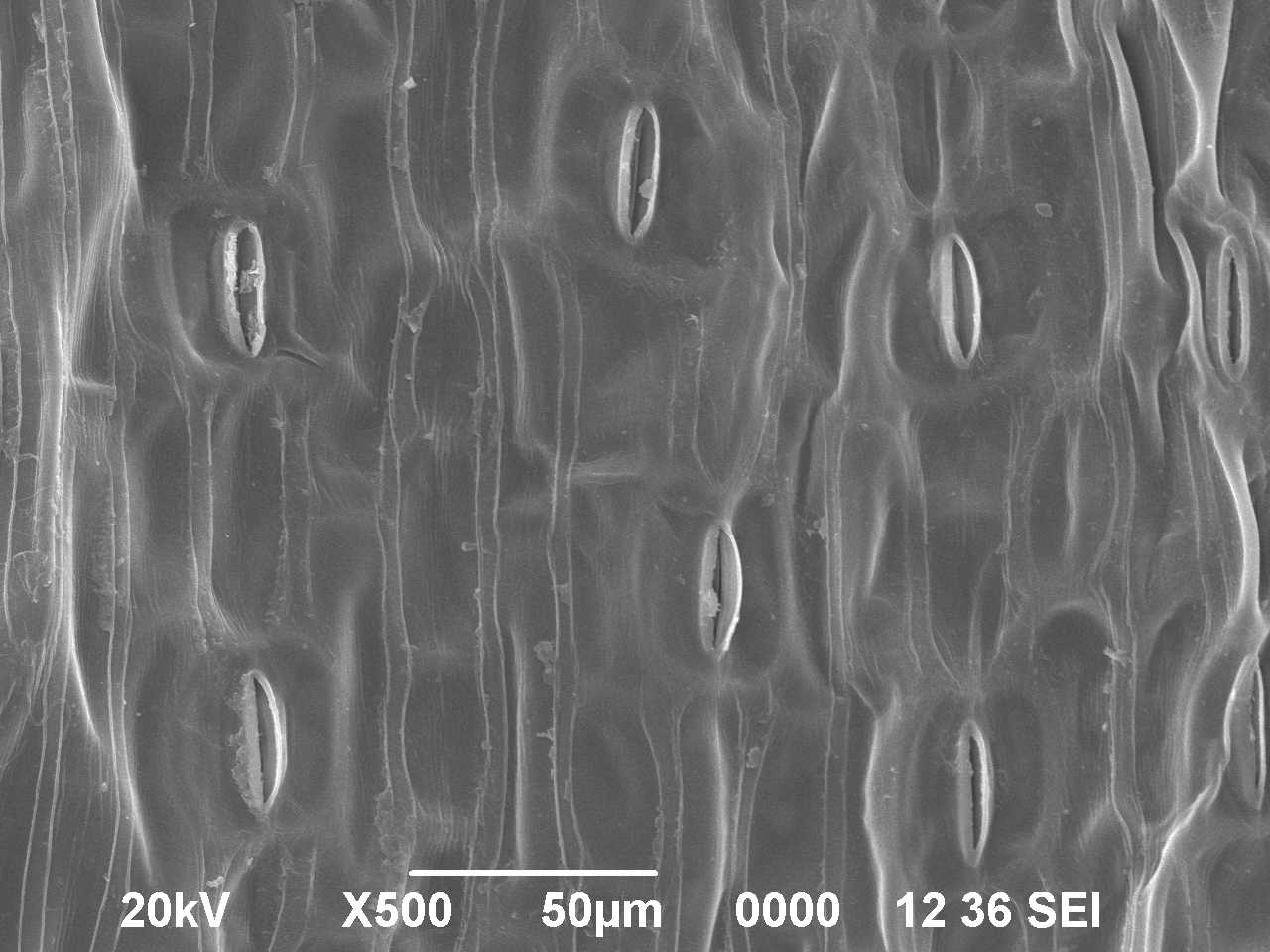

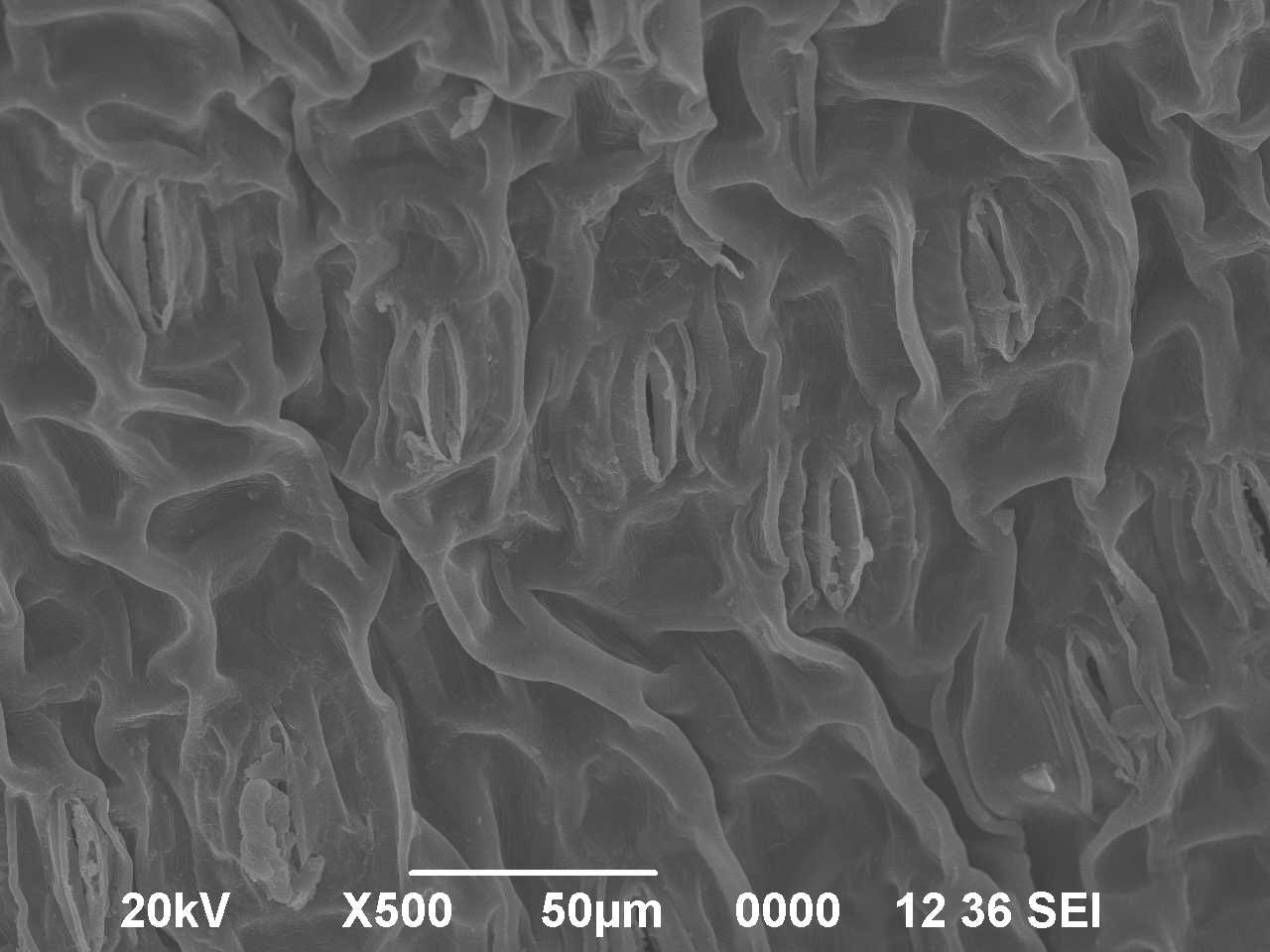


a

b

| Element | Atomic number | Series | Weight %  (normalized) | Atomic % |
| --- | --- | --- | --- | --- |
| C | 6 | K | 47.84 | 54.23 |
| N | 7 | K | 14.05 | 13.66 |
| O | 8 | K | 37.49 | 31.91 |
| Na | 11 | K | 0.08 | 0.05 |
| P | 15 | K | 0.13 | 0.06 |
| S | 16 | K | 0.04 | 0.02 |
| K | 19 | K | 0.01 | 0.00 |
| Ca | 20 | K | 0.02 | 0.01 |
| Fe | 26 | K | 0.06 | 0.01 |
| Cu | 29 | K | 0.14 | 0.03 |
| Zn | 30 | K | 0.16 | 0.03 |
| Total |  |  | 100 | 100 |


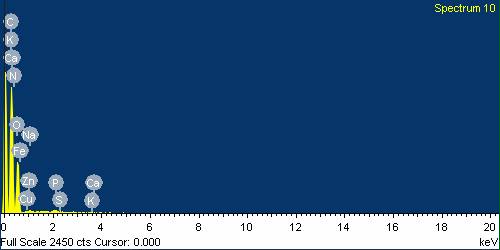


c

| Element | Atomic number | Series | Weight %  (normalized) | Atomic % |
| --- | --- | --- | --- | --- |
| C | 6 | K | 38.87 | 45.60 |
| N | 7 | K | 12.66 | 12.74 |
| O | 8 | K | 46.00 | 40.50 |
| Na | 11 | K | 1.36 | 0.84 |
| Mg | 12 | K | 0.23 | 0.14 |
| P | 15 | K | 0.06 | 0.03 |
| S | 16 | K | 0.04 | 0.02 |
| K | 19 | K | 0.04 | 0.01 |
| Ca | 20 | K | 0.19 | 0.07 |
| Cu | 29 | K | 0.06 | 0.01 |
| Sb | 51 | L | 0.50 | 0.06 |
| Total |  |  | 100 | 100 |


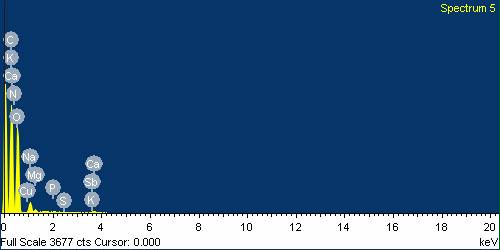


d

**Supplementary Fig. 2**

| Element | Atomic number | Series | Weight %  (normalized) | Atomic % |
| --- | --- | --- | --- | --- |
| C | 6 | K | 44.73 | 51.52 |
| N | 7 | K | 9.72 | 9.60 |
| O | 8 | K | 44.09 | 38.12 |
| Na | 11 | K | 0.86 | 0.52 |
| Mg | 12 | K | 0.09 | 0.05 |
| P | 15 | K | 0.09 | 0.04 |
| S | 16 | K | 0.10 | 0.04 |
| K | 19 | K | 0.13 | 0.05 |
| Ca | 20 | K | 0.11 | 0.04 |
| Fe | 26 | K | 0.08 | 0.02 |
| Total |  |  | 100 | 100 |


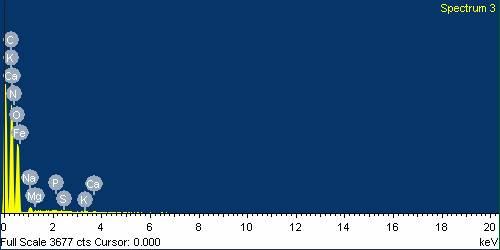


a

| Element | Atomic number | Series | Weight %  (normalized) | Atomic % |
| --- | --- | --- | --- | --- |
| C | 6 | K | 44.75 | 51.57 |
| N | 7 | K | 9.70 | 9.59 |
| O | 8 | K | 43.88 | 38.12 |
| Na | 11 | K | 1.03 | 0.62 |
| Mg | 12 | K | 0.09 | 0.05 |
| P | 15 | K | 0.09 | 0.04 |
| S | 16 | K | 0.10 | 0.04 |
| K | 19 | K | 0.13 | 0.05 |
| Ca | 20 | K | 0.11 | 0.04 |
| Fe | 26 | K | 0.08 | 0.02 |
| Zn | 30 | K | 0.04 | 0.01 |
| Total |  |  | 100 | 100 |


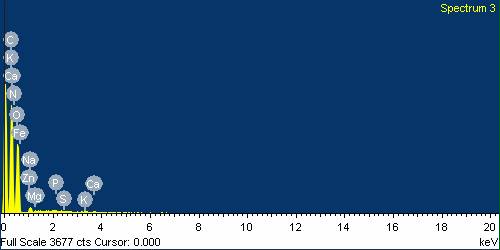


b

| Element | Atomic number | Series | Weight %  (normalized) | Atomic % |
| --- | --- | --- | --- | --- |
| C | 6 | K | 41.83 | 48.47 |
| N | 7 | K | 12.66 | 12.58 |
| O | 8 | K | 43.97 | 38.25 |
| Na | 11 | K | 0.80 | 0.48 |
| P | 15 | K | 0.10 | 0.04 |
| S | 16 | K | 0.08 | 0.04 |
| K | 19 | K | 0.08 | 0.03 |
| Ca | 20 | K | 0.07 | 0.03 |
| Fe | 26 | K | 0.02 | 0.00 |
| Cu | 29 | K | 0.14 | 0.03 |
| Zn | 30 | K | 0.26 | 0.06 |
| Total |  |  | 100 | 100 |


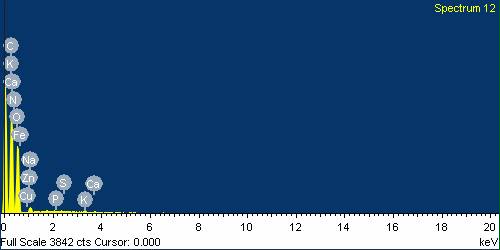


c

**Supplementary Fig. 3**

| Element | Atomic number | Series | Weight %  (normalized) | Atomic % |
| --- | --- | --- | --- | --- |
| C | 6 | K | 49.33 | 57.36 |
| N | 7 | K | 10.36 | 10.33 |
| O | 8 | K | 35.63 | 31.10 |
| Na | 11 | K | 0.54 | 0.33 |
| P | 15 | K | 0.01 | 0.01 |
| S | 16 | K | 0.09 | 0.04 |
| K | 19 | K | 0.26 | 0.09 |
| Ca | 20 | K | 0.41 | 0.14 |
| Fe | 26 | K | 0.06 | 0.02 |
| Cu | 29 | K | 0.79 | 0.17 |
| Zn | 30 | K | 1.31 | 0.28 |
| Sb | 51 | L | 1.20 | 0.14 |
| Total |  |  | 100 | 100 |

**
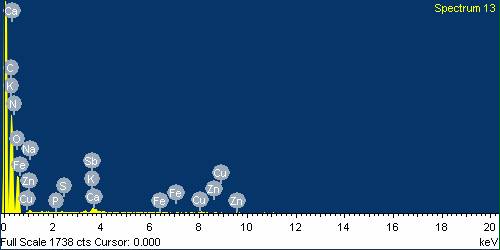
**

a

| Element | Atomic number | Series | Weight %  (normalized) | Atomic % |
| --- | --- | --- | --- | --- |
| C | 6 | K | 40.97 | 48.74 |
| N | 7 | K | 10.35 | 10.56 |
| O | 8 | K | 43.84 | 39.15 |
| Na | 11 | K | 1.38 | 0.86 |
| Mg | 12 | K | 0.08 | 0.05 |
| P | 15 | K | 0.05 | 0.02 |
| S | 16 | K | 0.04 | 0.02 |
| K | 19 | K | 0.24 | 0.09 |
| Ca | 20 | K | 0.29 | 0.11 |
| Fe | 26 | K | 0.11 | 0.03 |
| Cu | 29 | K | 0.32 | 0.07 |
| Zn | 30 | K | 0.36 | 0.08 |
| Sb | 51 | L | 1.97 | 0.23 |
| Total |  |  | 100 | 100 |

**
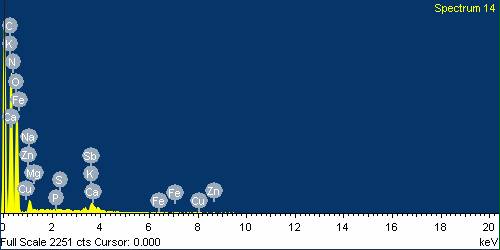
**

b

| Element | Atomic number | Series | Weight %  (normalized) | Atomic % |
| --- | --- | --- | --- | --- |
| C | 6 | K | 51.08 | 58.14 |
| N | 7 | K | 9.19 | 8.97 |
| O | 8 | K | 37.43 | 31.98 |
| Na | 11 | K | 0.76 | 0.45 |
| Mg | 12 | K | 0.07 | 0.04 |
| P | 15 | K | 0.15 | 0.07 |
| S | 16 | K | 0.32 | 0.14 |
| K | 19 | K | 0.20 | 0.07 |
| Ca | 20 | K | 0.16 | 0.05 |
| Fe | 26 | K | 0.14 | 0.04 |
| Zn | 30 | K | 0.05 | 0.01 |
| Sb | 51 | L | 0.45 | 0.05 |
| Total |  |  | 100 | 100 |

**
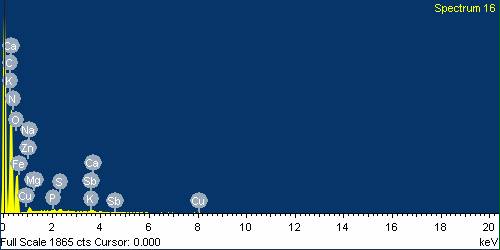
**

c

**Supplementary Fig. 4**

**
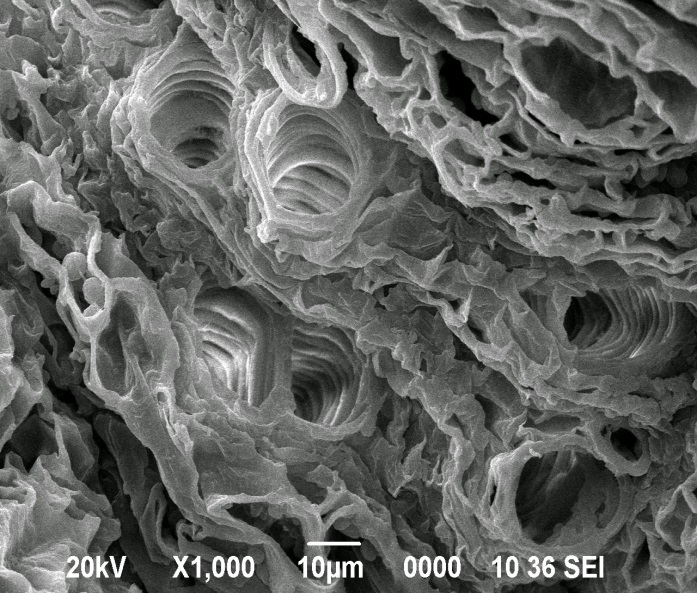

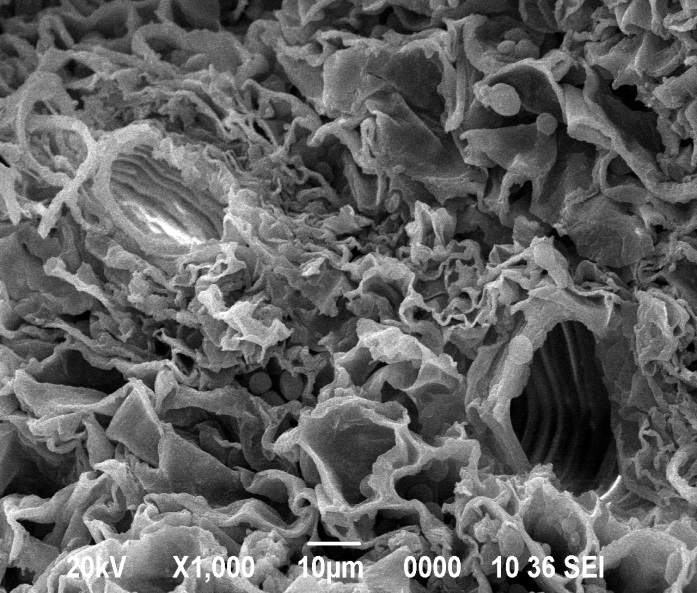
**

b

a

**Supplementary Fig. 5**

| Element | Atomic number | Series | Weight %  (normalized) | Atomic % |
| --- | --- | --- | --- | --- |
| C | 6 | K | 48.31 | 55.53 |
| N | 7 | K | 6.55 | 6.45 |
| O | 8 | K | 43.23 | 37.31 |
| Na | 11 | K | 0.38 | 0.23 |
| Mg | 12 | K | 0.17 | 0.10 |
| P | 15 | K | 0.10 | 0.05 |
| S | 16 | K | 0.10 | 0.04 |
| K | 19 | K | 0.20 | 0.07 |
| Ca | 20 | K | 0.31 | 0.11 |
| Fe | 26 | K | 0.20 | 0.05 |
| Cu | 29 | K | 0.37 | 0.08 |
| Zn | 30 | K | 0.27 | 0.06 |
| Total |  |  | 100 | 100 |

**
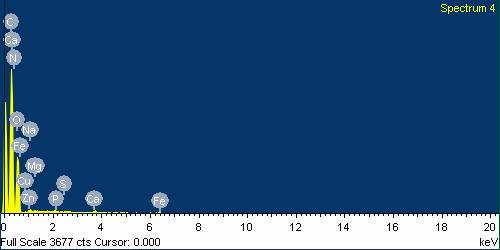
**

a

| Element | Atomic number | Series | Weight %  (normalized) | Atomic % |
| --- | --- | --- | --- | --- |
| C | 6 | K | 48.26 | 55.35 |
| N | 7 | K | 6.58 | 6.47 |
| O | 8 | K | 43.59 | 37.53 |
| Na | 11 | K | 0.42 | 0.25 |
| Mg | 12 | K | 0.17 | 0.10 |
| P | 15 | K | 0.10 | 0.05 |
| S | 16 | K | 0.10 | 0.04 |
| K | 19 | K | 0.20 | 0.07 |
| Ca | 20 | K | 0.32 | 0.11 |
| Fe | 26 | K | 0.20 | 0.05 |
| Zn | 30 | K | 0.25 | 0.05 |
| Total |  |  | 100 | 100 |

**
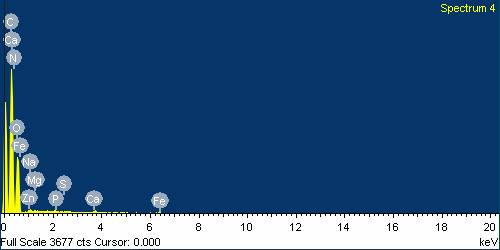
**

b

**Supplementary Fig. 6**

| Element | Atomic number | Series | Weight %  (normalized) | Atomic % |
| --- | --- | --- | --- | --- |
| C | 6 | K | 58.59 | 66.24 |
| N | 7 | K | 6.74 | 6.54 |
| O | 8 | K | 30.99 | 26.31 |
| Na | 11 | K | 0.45 | 0.27 |
| Mg | 12 | K | 0.13 | 0.07 |
| P | 15 | K | 0.11 | 0.05 |
| K | 19 | K | 0.19 | 0.07 |
| Ca | 20 | K | 0.14 | 0.05 |
| Fe | 26 | K | 0.10 | 0.02 |
| Cu | 29 | K | 0.67 | 0.14 |
| Zn | 30 | K | 0.34 | 0.07 |
| Sb | 51 | L | 1.55 | 0.17 |
| Total |  |  | 100 | 100 |

**
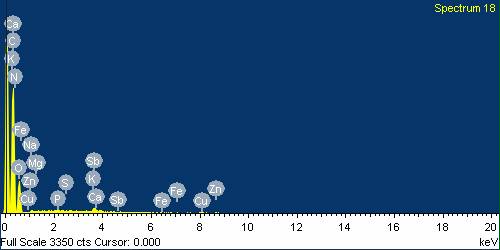
**

a

| Element | Atomic number | Series | Weight %  (normalized) | Atomic % |
| --- | --- | --- | --- | --- |
| C | 6 | K | 45.49 | 52.39 |
| N | 7 | K | 11.93 | 11.78 |
| O | 8 | K | 40.57 | 35.08 |
| Na | 11 | K | 0.65 | 0.39 |
| Mg | 12 | K | 0.23 | 0.13 |
| S | 16 | K | 0.02 | 0.01 |
| K | 19 | K | 0.07 | 0.03 |
| Ca | 20 | K | 0.17 | 0.06 |
| Cu | 29 | K | 0.18 | 0.04 |
| Zn | 30 | K | 0.17 | 0.04 |
| Sb | 51 | L | 0.52 | 0.06 |
| Total |  |  | 100 | 100 |

**
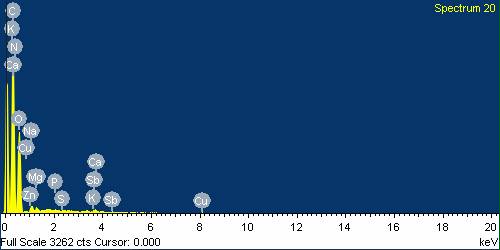
**

b

| Element | Atomic number | Series | Weight %  (normalized) | Atomic % |
| --- | --- | --- | --- | --- |
| C | 6 | K | 54.44 | 61.36 |
| N | 7 | K | 6.76 | 6.54 |
| O | 8 | K | 37.23 | 31.50 |
| Na | 11 | K | 0.51 | 0.30 |
| Mg | 12 | K | 0.10 | 0.05 |
| P | 15 | K | 0.09 | 0.04 |
| S | 16 | K | 0.07 | 0.03 |
| K | 19 | K | 0.08 | 0.03 |
| Ca | 20 | K | 0.09 | 0.03 |
| Cu | 29 | K | 0.28 | 0.06 |
| Zn | 30 | K | 0.15 | 0.03 |
| Sb | 51 | L | 0.12 | 0.01 |
| Total |  |  | 100 | 100 |

**
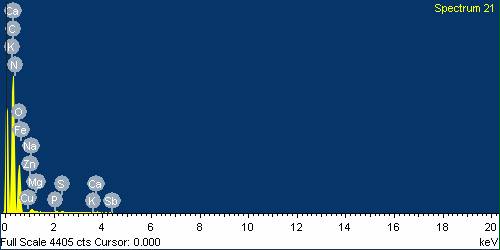
**

b

**Supplementary Fig. 7**


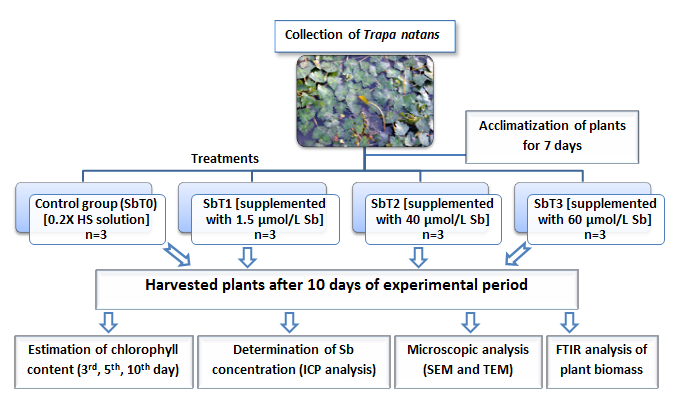

Supplement: Supplementary file 1 — Supplementary Figures. [file 41598_2021_89865_MOESM1_ESM.docx]
