# Supplementary material for: Antimony induced structural and ultrastructural changes in Trapa natans
Source: Sci Rep. 2021 May 21;11:10695. doi: 10.1038/s41598-021-89865-2 (PMC8140150; doi:10.1038/s41598-021-89865-2)
Supplement: Supplementary file 2 — Supplementary Tables. [file 41598_2021_89865_MOESM2_ESM.docx]

**Antimony Induced Structural and Ultrastructural Changes in *Trapa natans***

**­­**Sangita Baruah^1^**·** Monashree Sarma Bora^1^**·** Sanghita Dutta^1^**·** Kalyan Kumar Hazarika^2^**·** Pronab Mudoi^2^**·** Kali Prasad Sarma^1^*

1. Department of Environmental Science, Tezpur University, Napaam, Tezpur, Assam, India
2. Department of Molecular Biology and Biotechnology, Tezpur University, Napaam, Tezpur, Assam, India

Corresponding author: [sarmakp@tezu.ernet.in](mailto:sarmakp@tezu.ernet.in)

Sangita Baruah and Monashree Sarma Bora are co-first author (Both of them contributed equally to this work)

**List of Supplementary Table Captions**

**Supplementary Table 1.** One way ANOVA results of total chlorophyll content in *T. natans* under Sb exposure.

**Supplementary Table 2.** Tukey HSD test results of total chlorophyll content in *T. natans* under Sb exposure.

**Supplementary Table 3.** One way ANOVA results of Sb accumulation in different vegetative tissues of *T. natans.*

**Supplementary Table 4.** Tukey HSD test results of Sb accumulation in different vegetative tissues of *T. natans*

**Supplementary Table 5.** One way ANOVA post hoc test (Tukey HSD) results of Sb accumulation in *T. natans* under different Sb treatments.

**Supplementary Table 6.** Tukey HSD test results of Sb accumulation in *T. natans* under different Sb treatments.

**Supplementary Table 1**

|  | | Sum of Squares | df | Mean Square | F | Sig. |  |
| --- | --- | --- | --- | --- | --- | --- | --- |
| 3^rd^ day | Between Groups | .288 | 3 | .096 | 548.889 | .000 |  |
|  | Within Groups | .001 | 8 | .000 |  |  |  |
|  | Total | .290 | 11 |  |  |  |  |
| 5^th^ day | Between Groups | .332 | 3 | .111 | 699.158 | .000 |  |
|  | Within Groups | .001 | 8 | .000 |  |  |  |
|  | Total | .333 | 11 |  |  |  |  |
| 10^th^ day | Between Groups | .510 | 3 | .170 | 971.429 | .000 |  |
|  | Within Groups | .001 | 8 | .000 |  |  |  |
|  | Total | .511 | 11 |  |  |  |  |
| *. The mean difference is significant at the 0.05 level. | | | | | | | |

**Supplementary Table 2**

| Dependent Variable | (I) Group | (J) Group | Mean Difference (I-J) | Std. Error | Sig. | 95% Confidence Interval | |
| --- | --- | --- | --- | --- | --- | --- | --- |
|  |  |  |  |  |  | Lower Bound | Upper Bound |
| 3^rd^ day | Control | SbT1 | .10333^*^ | .01080 | .000 | .0687 | .1379 |
|  |  | SbT2 | .29667^*^ | .01080 | .000 | .2621 | .3313 |
|  |  | SbT3 | .39333^*^ | .01080 | .000 | .3587 | .4279 |
|  | SbT1 | Control | -.10333^*^ | .01080 | .000 | -.1379 | -.0687 |
|  |  | SbT2 | .19333^*^ | .01080 | .000 | .1587 | .2279 |
|  |  | SbT3 | .29000^*^ | .01080 | .000 | .2554 | .3246 |
|  | SbT2 | Control | -.29667^*^ | .01080 | .000 | -.3313 | -.2621 |
|  |  | SbT1 | -.19333^*^ | .01080 | .000 | -.2279 | -.1587 |
|  |  | SbT3 | .09667^*^ | .01080 | .000 | .0621 | .1313 |
|  | SbT3 | Control | -.39333^*^ | .01080 | .000 | -.4279 | -.3587 |
|  |  | SbT1 | -.29000^*^ | .01080 | .000 | -.3246 | -.2554 |
|  |  | SbT2 | -.09667^*^ | .01080 | .000 | -.1313 | -.0621 |
| 5^th^ day | Control | SbT1 | .19333^*^ | .01027 | .000 | .1604 | .2262 |
|  |  | SbT2 | .40000^*^ | .01027 | .000 | .3671 | .4329 |
|  |  | SbT3 | .40000^*^ | .01027 | .000 | .3671 | .4329 |
|  | SbT1 | Control | -.19333^*^ | .01027 | .000 | -.2262 | -.1604 |
|  |  | SbT2 | .20667^*^ | .01027 | .000 | .1738 | .2396 |
|  |  | SbT3 | .20667^*^ | .01027 | .000 | .1738 | .2396 |
|  | SbT2 | Control | -.40000^*^ | .01027 | .000 | -.4329 | -.3671 |
|  |  | SbT1 | -.20667^*^ | .01027 | .000 | -.2396 | -.1738 |
|  |  | SbT3 | .00000 | .01027 | 1.000 | -.0329 | .0329 |
|  | SbT3 | Control | -.40000^*^ | .01027 | .000 | -.4329 | -.3671 |
|  |  | SbT1 | -.20667^*^ | .01027 | .000 | -.2396 | -.1738 |
|  |  | SbT2 | .00000 | .01027 | 1.000 | -.0329 | .0329 |
| 10^th^ day | Control | SbT1 | .40000^*^ | .01080 | .000 | .3654 | .4346 |
|  |  | SbT2 | .50000^*^ | .01080 | .000 | .4654 | .5346 |
|  |  | SbT3 | .50000^*^ | .01080 | .000 | .4654 | .5346 |
|  | SbT1 | Control | -.40000^*^ | .01080 | .000 | -.4346 | -.3654 |
|  |  | SbT2 | .10000^*^ | .01080 | .000 | .0654 | .1346 |
|  |  | SbT3 | .10000^*^ | .01080 | .000 | .0654 | .1346 |
|  | SbT2 | Control | -.50000^*^ | .01080 | .000 | -.5346 | -.4654 |
|  |  | SbT1 | -.10000^*^ | .01080 | .000 | -.1346 | -.0654 |
|  |  | SbT3 | .00000 | .01080 | 1.000 | -.0346 | .0346 |
|  | SbT3 | Control | -.50000^*^ | .01080 | .000 | -.5346 | -.4654 |
|  |  | SbT1 | -.10000^*^ | .01080 | .000 | -.1346 | -.0654 |
|  |  | SbT2 | .00000 | .01080 | 1.000 | -.0346 | .0346 |
| *. The mean difference is significant at the 0.05 level. | | | | | | | |

**Supplementary Table 3**

|  | | Sum of Squares | df | Mean Square | F | Sig. |
| --- | --- | --- | --- | --- | --- | --- |
| Leaf | Between Groups | 601.234 | 2 | 300.617 | 31.110 | .001 |
|  | Within Groups | 57.978 | 6 | 9.663 |  |  |
|  | Total | 659.212 | 8 |  |  |  |
| Stem | Between Groups | 2110.878 | 2 | 1055.439 | 104.789 | .000 |
|  | Within Groups | 60.432 | 6 | 10.072 |  |  |
|  | Total | 2171.310 | 8 |  |  |  |
| Root | Between Groups | 1153.061 | 2 | 576.530 | 50.363 | .000 |
|  | Within Groups | 68.685 | 6 | 11.448 |  |  |
|  | Total | 1221.746 | 8 |  |  |  |

*. The mean difference is significant at the 0.05 level.

**Supplementary Table 4**

| Dependent Variable | (I)  Treatment | (J)  Treatment | Mean Difference (I-J) | Std. Error | Sig. | 95% Confidence Interval | |
| --- | --- | --- | --- | --- | --- | --- | --- |
|  |  |  |  |  |  | Lower Bound | Upper Bound |
| Leaf | SbT1 | SbT2 | -18.85000^*^ | 2.53811 | .001 | -26.6376 | -11.0624 |
|  |  | SbT3 | -3.58333 | 2.53811 | .393 | -11.3709 | 4.2043 |
|  | SbT2 | SbT1 | 18.85000^*^ | 2.53811 | .001 | 11.0624 | 26.6376 |
|  |  | SbT3 | 15.26667^*^ | 2.53811 | .002 | 7.4791 | 23.0543 |
|  | SbT3 | SbT1 | 3.58333 | 2.53811 | .393 | -4.2043 | 11.3709 |
|  |  | SbT2 | -15.26667^*^ | 2.53811 | .002 | -23.0543 | -7.4791 |
| Stem | SbT1 | SbT2 | -2.26333 | 2.59127 | .675 | -10.2141 | 5.6874 |
|  |  | SbT3 | 31.29667^*^ | 2.59127 | .000 | 23.3459 | 39.2474 |
|  | SbT2 | SbT1 | 2.26333 | 2.59127 | .675 | -5.6874 | 10.2141 |
|  |  | SbT3 | 33.56000^*^ | 2.59127 | .000 | 25.6093 | 41.5107 |
|  | SbT3 | SbT1 | -31.29667^*^ | 2.59127 | .000 | -39.2474 | -23.3459 |
|  |  | SbT2 | -33.56000^*^ | 2.59127 | .000 | -41.5107 | -25.6093 |
| Root | SbT1 | SbT2 | -27.72333^*^ | 2.76255 | .000 | -36.1996 | -19.2471 |
|  |  | SbT3 | -13.55667^*^ | 2.76255 | .006 | -22.0329 | -5.0804 |
|  | SbT2 | SbT1 | 27.72333^*^ | 2.76255 | .000 | 19.2471 | 36.1996 |
|  |  | SbT3 | 14.16667^*^ | 2.76255 | .005 | 5.6904 | 22.6429 |
|  | SbT3 | SbT1 | 13.55667^*^ | 2.76255 | .006 | 5.0804 | 22.0329 |
|  |  | SbT2 | -14.16667^*^ | 2.76255 | .005 | -22.6429 | -5.6904 |
| *. The mean difference is significant at the 0.05 level. | | | | | | | |

**Supplementary Table 5**

|  | | Sum of Squares | df | Mean Square | F | Sig. |
| --- | --- | --- | --- | --- | --- | --- |
| SbT1 | Between Groups | 109.143 | 2 | 54.571 | 4.977 | .053 |
|  | Within Groups | 65.794 | 6 | 10.966 |  |  |
|  | Total | 174.936 | 8 |  |  |  |
| SbT2 | Between Groups | 845.470 | 2 | 422.735 | 37.892 | .000 |
|  | Within Groups | 66.937 | 6 | 11.156 |  |  |
|  | Total | 912.407 | 8 |  |  |  |
| SbT3 | Between Groups | 2806.478 | 2 | 1403.239 | 154.872 | .000 |
|  | Within Groups | 54.364 | 6 | 9.061 |  |  |
|  | Total | 2860.842 | 8 |  |  |  |

*. The mean difference is significant at the 0.05 level.

**Supplementary Table 6**

| Dependent Variable | (I) Group | (J) Group | Mean Difference (I-J) | Std. Error | Sig. | 95% Confidence Interval | |
| --- | --- | --- | --- | --- | --- | --- | --- |
|  |  |  |  |  |  | Lower Bound | Upper Bound |
| SbT1 | Leaf | Stem | -8.19000 | 2.70378 | .052 | -16.4859 | .1059 |
|  |  | Root | -6.16000 | 2.70378 | .135 | -14.4559 | 2.1359 |
|  | Stem | Leaf | 8.19000 | 2.70378 | .052 | -.1059 | 16.4859 |
|  |  | Root | 2.03000 | 2.70378 | .744 | -6.2659 | 10.3259 |
|  | Root | Leaf | 6.16000 | 2.70378 | .135 | -2.1359 | 14.4559 |
|  |  | Stem | -2.03000 | 2.70378 | .744 | -10.3259 | 6.2659 |
| SbT2 | Leaf | Stem | 8.39667^*^ | 2.72718 | .049 | .0289 | 16.7644 |
|  |  | Root | -15.03333^*^ | 2.72718 | .004 | -23.4011 | -6.6656 |
|  | Stem | Leaf | -8.39667^*^ | 2.72718 | .049 | -16.7644 | -.0289 |
|  |  | Root | -23.43000^*^ | 2.72718 | .000 | -31.7977 | -15.0623 |
|  | Root | Leaf | 15.03333^*^ | 2.72718 | .004 | 6.6656 | 23.4011 |
|  |  | Stem | 23.43000^*^ | 2.72718 | .000 | 15.0623 | 31.7977 |
| SbT3 | Leaf | Stem | 26.69000^*^ | 2.45773 | .000 | 19.1490 | 34.2310 |
|  |  | Root | -16.13333^*^ | 2.45773 | .001 | -23.6743 | -8.5923 |
|  | Stem | Leaf | -26.69000^*^ | 2.45773 | .000 | -34.2310 | -19.1490 |
|  |  | Root | -42.82333^*^ | 2.45773 | .000 | -50.3643 | -35.2823 |
|  | Root | Leaf | 16.13333^*^ | 2.45773 | .001 | 8.5923 | 23.6743 |
|  |  | Stem | 42.82333^*^ | 2.45773 | .000 | 35.2823 | 50.3643 |
| *. The mean difference is significant at the 0.05 level. | | | | | | | |
